# Oocyte‐specific deletion of eukaryotic translation initiation factor 5 causes apoptosis of mouse oocytes within the early‐growing follicles by mitochondrial fission defect‐reactive oxygen species‐DNA damage

**DOI:** 10.1002/ctm2.1791

**Published:** 2024-08-07

**Authors:** Weiyong Wang, Huiyu Liu, Shuang Liu, Tiantian Hao, Ying Wei, Hongwei Wei, Wenjun Zhou, Xiaodan Zhang, Xiaoqiong Hao, Meijia Zhang

**Affiliations:** ^1^ The Innovation Centre of Ministry of Education for Development and Diseases the Second Affiliated Hospital School of Medicine South China University of Technology Guangzhou China; ^2^ Department of Physiology Baotou Medical College Baotou China

**Keywords:** eIF5, follicle development, mitochondrial fission, oocyte growth, POI

## Abstract

**Background:**

Mutations in several translation initiation factors are closely associated with premature ovarian insufficiency (POI), but the underlying pathogenesis remains largely unknown.

**Methods and results:**

We generated eukaryotic translation initiation factor 5 (*Eif5*) conditional knockout mice aiming to investigate the function of eIF5 during oocyte growth and follicle development. Here, we demonstrated that *Eif5* deletion in mouse primordial and growing oocytes both resulted in the apoptosis of oocytes within the early‐growing follicles. Further studies revealed that *Eif5* deletion in oocytes downregulated the levels of mitochondrial fission‐related proteins (p‐DRP1, FIS1, MFF and MTFR) and upregulated the levels of the integrated stress response‐related proteins (AARS1, SHMT2 and SLC7A1) and genes (*Atf4*, *Ddit3* and *Fgf21*). Consistent with this, *Eif5* deletion in oocytes resulted in mitochondrial dysfunction characterized by elongated form, aggregated distribution beneath the oocyte membrane, decreased adenosine triphosphate content and mtDNA copy numbers, and excessive accumulation of reactive oxygen species (ROS) and mitochondrial superoxide. Meanwhile, *Eif5* deletion in oocytes led to a significant increase in the levels of DNA damage response proteins (γH2AX, p‐CHK2 and p‐p53) and proapoptotic proteins (PUMA and BAX), as well as a significant decrease in the levels of anti‐apoptotic protein BCL‐xL.

**Conclusion:**

These findings indicate that *Eif5* deletion in mouse oocytes results in the apoptosis of oocytes within the early‐growing follicles via mitochondrial fission defects, excessive ROS accumulation and DNA damage. This study provides new insights into pathogenesis, genetic diagnosis and potential therapeutic targets for POI.

**Key points:**

- *Eif5* deletion in oocytes leads to arrest in oocyte growth and follicle development.
- *Eif5* deletion in oocytes impairs the translation of mitochondrial fission‐related proteins, followed by mitochondrial dysfunction.
- Depletion of *Eif5* causes oocyte apoptosis via ROS accumulation and DNA damage response pathway.

## Graphical abstract

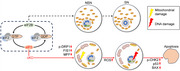

## 1 INTRODUCTION

The nonrenewable primordial follicle pool is established in mammals before or around birth.^1^ The primordial follicles are maintained in a dormant state with low transcriptional and translational activities.^2^ A minority of primordial follicles are activated during each follicular wave and undergo orderly development to provide mature oocytes, thereby contributing to female fertility.^3^ The activated oocytes enter a growth phase characterized by a massive increase in volume, mRNA transcription, protein synthesis and cytoplasmic organelle biogenesis.^2^, ^4^ Dramatic mitochondrial biogenesis through mtDNA replication and mitochondrial fission provides adenosine triphosphate (ATP) for protein translation and gene transcription during oocyte growth.^5^, ^6^, ^7^ Concomitantly, efficient translation of oocyte‐secreted factors promotes the proliferation and metabolism of granulosa cells, which in turn provide essential pyruvates, amino acids and steroids for oocyte growth and meiotic maturation.^8^, ^9^ The bidirectional communication between granulosa cells and oocytes relies on gap junctions and transzonal projections (TZPs).^10^ When the chromatin configuration changes from the non‐surrounded nucleolus (NSN) to the surrounded nucleolus (SN), oocytes undergo transcriptional silencing and acquire the competence of meiotic resumption. Subsequently, oocyte maturation completely depends on the translation control.^11^, ^12^, ^13^ Thus, spatiotemporal regulation of protein translation is indispensable for oocyte growth and meiotic maturation.

Noteworthy, the mutations in translation initiation‐related genes are closely associated with premature ovarian insufficiency (POI), such as eukaryotic translation initiation factor (*EIF*) *2B2*, *EIF2B4*, *EIF2B5*, EIF1A X‐linked (*EIF1AX*) and EIF4E nuclear import factor (*EIF4ENIF1*).^14^, ^15^ However, the underlying pathogenesis of POI caused by translation‐related gene mutations remains largely unknown. As a nucleocytoplasmic shuttle protein, eIF4ENIF1 maintains translational homeostasis by regulating the transport of eIF4E.^16^ *Eif4enif1* haploinsufficiency causes mitochondrial hyperfusion by disturbing the translation of mitochondrial fission and fusion proteins, resulting in female mouse subfertility.^17^ Similar to canonical eIF4E, germ cell‐specific eIF4E family member 1B (eIF4E1B) recognizes the cap‐structure of mRNA and participates in protein translation initiation.^18^ *Eif4e1b* deletion in mouse oocytes causes embryonic development arrest by impairing the selective translation of maternal mRNA essential for oocyte‐to‐embryo transition and zygotic genome activation.^18^, ^19^ Furthermore, several factors also participate in translation initiation. The mammalian target of rapamycin (mTOR) promotes translation initiation by phosphorylating eIF4E binding protein 1 (eIF4EBP1).^20^ Ribosomal protein S26 (RPS26) is a component of ribosomes that is required for translation initiation.^21^ *Mtor* deletion in mouse oocytes impairs oocyte maturation by downregulating the translation of proteins essential for spindle assembly and mRNA metabolism.^22^ *Rps26* deletion in mouse oocytes causes an arrest in oocyte growth and follicle development by downregulating the translation of oocyte‐secreted factors and gap junction proteins.^23^ Collectively, this evidence implies that deficiencies in different translational regulators lead to different reproductive phenotypes.

eIF5 regulates translation initiation by inactivating the eIF2·GTP·Met‐tRNAi ternary complex (TC).^24^ During each round of translation, TC is recruited to the 40S ribosomal subunit to form the 43S preinitiation complex (PIC).^25^ 43S‐PIC subsequently binds to the capped 5′ end of mRNA and scans the leader sequence to search for an AUG start codon, thereby initiating translation.^26^ After starting codon recognition, eIF5 stimulates the hydrolysis of eIF2‐bound GTP to GDP by its GTPase accelerating protein activity, leading to the release of an inactive eIF2·GDP complex from the ribosomes.^27^ Meanwhile, eIF5 maintains the stability of inactive eIF2·GDP complex by its GDP dissociation inhibitor activity.^28^ For subsequent rounds of translation initiation, the inactive eIF2·GDP is recycled to an active eIF2·GTP by eIF2B.^29^ The knockdown of *Eif5* in mouse embryonic fibroblast cells causes suppression of partial protein translation but has no effects on stress‐induced proteins.^30^ The overexpression of *Eif5* in mouse zygotes impairs embryo compaction and blastocyst formation by suppressing global protein translation and increasing non‐AUG initiation.^31^ However, the role of eIF5 in oocyte growth and follicle development remains unclear.

Here, the effects of eIF5 on mammalian oocyte growth and follicle development were revealed by conditionally deleting *Eif5* in mouse primordial and growing oocytes. The findings indicate that the *Eif5* deletion in mouse oocytes results in the apoptosis of oocytes within the early‐growing follicles. These observed phenotypes are attributed to impaired translation of mitochondrial fission‐related proteins, subsequent excessive ROS accumulation and DNA damage.

## 2 MATERIALS AND METHODS

### 2.1 Animals

All wild‐type (WT) mice were procured from Guangdong Medical Laboratory Animal Center. *Eif5* ^flox/flox^ mice were generated by Shanghai Model Organisms Center, Inc. through homologous recombination (HR) of fertilized eggs. Mice with oocyte‐specific ablation of *Eif5* in primordial and primary oocytes were generated by mating *Eif5* ^flox/flox^ female mice with *Gdf9*‐Cre (hereafter referred to as GcKO) and *Zp3*‐Cre (hereafter referred to as ZcKO) male mice, respectively. Polymerase chain reaction (PCR) was used for genotype identification, and all specific primers are provided in Table S1. All animal protocols were approved by the Institutional Animal Care and Use Committee of the South China University of Technology.

### 2.2 Histomorphological observation and follicle counting

The WT, GcKO and ZcKO ovaries were harvested and placed in 4% paraformaldehyde (PFA, Solarbio, P1110) for fixation. After this, the ovaries were paraffin‐embedded and sectioned serially at 5  µm. Periodic Acid Schiff (PAS) and Hematoxylin were used to stain the ovarian sections. Subsequently, the sections were installed in neutral resin for histomorphological examination. To quantify the number of follicles, the follicles were classified based on previously described methods.^18^ The total number of primordial follicles was calculated by multiplying the count in every fifth section by five, while the other stage follicles with clear nuclei of oocytes were quantified in every third section throughout the entire ovary.

### 2.3 Oocyte isolation and culture

WT and ZcKO mice at postnatal days (PD) 21−23 received intraperitoneal injections of 5 IU equine chorionic gonadotropin (eCG). After 48 h, the cumulus‐oocyte complexes (COCs) were released into the MEM medium. The germinal vesicle (GV) oocytes were then separated from the COCs and cultured in drops of M16 medium covered with mineral oil at 37°C. Germinal vesicle breakdown (GVBD) and the extrusion of the first polar body (PB1) were observed and recorded at 3 and 14 h, respectively. For superovulation and collection of metaphase II (MII)‐stage oocytes, the eCG‐primed mice were subsequently treated with 5 IU hCG (Sigma). After 13 h, the COCs were harvested from WT and ZcKO oviducts.

### 2.4 Transmission electron microscopy

To enrich the oocytes, WT and ZcKO GV oocytes were pre‐embedded in low melting point agarose after fixation with 2.5% glutaraldehyde. Subsequently, the oocytes were rinsed in phosphate‐buffered saline (PBS) and post‐fixed with 1% osmium acid for 2 h at 4°C. Following this, the oocytes underwent dehydration through gradient alcohol (50%, 60%, 70%, 80%, 90% and 100%) and 100% acetone. The dehydrated oocytes were then resin‐embedded to prepare 100 nm‐thick sections, which were further counterstained with 2% uranyl acetate and lead citrate, and images were finally captured using a transmission electron microscopy (TEM) (FEI, Tecnai G2 Spirit, 120 kV).

### 2.5 Immunofluorescence

The pre‐prepared ovarian sections were dewaxed and rehydrated using xylene and gradient ethanol, respectively. Subsequently, sections were subjected to 0.01 mol/L sodium citrate buffer (pH 6.0) for high‐temperature antigen retrieval, followed by blocking with 10% donkey serum for 1 h before incubating with primary antibodies overnight. After this, the sections were exposed to fluorescent secondary antibodies for 1 h at 37°C. Finally, nuclei were visualized using 4′, 6‐diamidino‐2‐phenylindole (DAPI) staining.

For immunofluorescence (IF) staining of oocytes, the collected oocytes were treated with 4% PFA and 0.25% Triton X‐100 for fixation and permeabilization, respectively. After blocking with 3% bovine serum albumin (BSA), oocytes were transferred to primary antibodies to incubate overnight at 4°C. After washing three times with PBS, the oocytes were exposed to fluorescent secondary antibodies for 1 h at 37°C. The nuclei were visualized with DAPI. All the sections and oocytes were submitted to an LSM 800 confocal microscope (Carl Zeiss) to capture images with the same parameters. The ZEN (Carl Zeiss, Version 3.1) was used to measure and quantify fluorescence intensity.

### 2.6 Bromodeoxyuridine incorporation assay

The WT and ZcKO mice at PD21 received intraperitoneal injections of 10 µL/g body weight of 10 mg/mL bromodeoxyuridine (BrdU) for 4 h. Subsequently, the collected ovaries underwent the above‐described procedures, including fixation, embedding, section preparation, antigen retrieval, blocking and antibody incubation to assess BrdU incorporation. Finally, the largest five sections of ovaries were selected for counting granulosa cells with BrdU‐positive signals.

### 2.7 In situ cell death detection

Ovarian cell apoptosis was assessed with a commercial kit (11684795910, Roche). Briefly, the dewaxed and rehydrated ovarian sections were permeabilized for 30 min at room temperature using proteinase K. Following two PBS rinses, these sections were treated with a TUNEL reaction mixture for 1 h at 37°C under dark conditions. Subsequently, the nuclei were visualized with DAPI and sections were analyzed directly using an LSM 800 confocal microscope.

### 2.8 Nascent protein and peptide assay

Nascent protein synthesis was assessed using the homopropargylglycine (HPG) incorporation. Specifically, the GV oocytes were first incubated in a Dulbecco′s Modified Eagle′s Medium without L‐methionine, enriched with 50 µM HPG for 30 min. Following two rinses with PBS containing 3% BSA, the oocytes were treated using 3.7% formaldehyde and 0.5% Triton X‐100 for fixation and permeabilization, respectively. The incorporation of HPG was visualized with commercial kits (C10429, Thermo Fisher), and then the nucleus was visualized using NuclearMask blue stain. All the oocytes were submitted to an LSM 800 confocal microscope to capture images.

Nascent peptide synthesis was assessed using the puromycin incorporation. Specifically, the WT and ZcKO mice at PD21 received intraperitoneal injections of 65 mg/kg puromycin. After 1.5 h, 200 GV oocytes were collected for Western blot analysis using a primary antibody of puromycin. The intensity of a total electrophoretic track reveals the ability of oocytes to synthesize nascent polypeptides.

### 2.9 Quantitative real‐time PCR

Note that, 100 GV oocytes from each WT, GcKO and ZcKO mice were used to extract total RNA using an RNeasy micro kit (74004, Qiagen), followed by synthesis of cDNA with QuantiTect reverse transcription kit (205311, Qiagen). The quantitative real‐time PCR (qRT‐PCR) was performed using SYBR Green PCR SuperMix (TransGen Biotech) on a LightCycler 96 system (Roche). The data were normalized by ribosomal protein L19 (*Rpl19*) and then the relative mRNA expression level was calculated via the 2^−∆∆Ct^ method. All qRT‐PCR primers are provided in Table S2.

### 2.10 RNA‐seq analysis

WT, GcKO and ZcKO GV oocytes mRNA libraries were submitted to the DNBSEQ platform with 100 bp paired‐end reads to sequence using Smart‐seq2 amplification. To obtain clean reads, we utilized SOAPnuke (v1.5.2) to filter out and eliminate original reads with low‐quality, primer and adapter incorporation, as well as unknown base N content exceeding 5%. Then the clean reads were aligned with the mouse reference genome GRCh38 by HISAT2 (v2.0.4). FPKM was calculated and standardized with RSEM (v1.2.8). Differential expression of genes between WT, GcKO and ZcKO was analyzed by an R package DESeq2, and the genes with q‐value ≤ .05 and Log_2_foldchange ≥ 1 were identified as candidate genes.

### 2.11 Western blot analysis

Note that, 100 GV oocytes were used to extract proteins using radioimmunoprecipitation assay lysis buffer. Denatured protein samples were separated using a sodium dodecyl‐sulfate polyacrylamide gel electrophoresis gel, and transferred to a polyvinylidene fluoride membrane. The membranes then underwent blocking with 5% skim milk and incubation overnight with primary antibodies listed in Table S3. Following washing with TBST, the hybridized membrane was exposed to secondary antibodies (1:5000, ZSGB‐BIO). Finally, the membranes were visualized with a Tanon 5200 imaging system using a Super sensitive ECL detection kit (ESL003, Biolight Biotech). All protein band densities were quantified using ImageJ software, with β‐actin serving as the internal control.

### 2.12 Quantitative analysis of single‐cell proteomics

WT and ZcKO mice at PD21‐23 received intraperitoneal injections of eCG. After 48 h, 600 GV oocytes (200 oocytes per sample) were collected from 6 mice of each genotype for proteomics analysis. Specifically, the oocytes were first lysed in 10 µL protein extraction buffer (8 M urea, 1% protease inhibitor cocktail) with non‐touching ultrasonic, and then protein was denatured at 95°C for 10 min. After natural cooling, the protein solution was digested in 20 ng/µL tryptase at 37°C overnight. Subsequently, the solution was reduced with 5 mM dithiothreitol at 56°C for 30 min and alkylated with 11 mM iodoacetamide at room temperature for 15 min in darkness. Ulteriorly, the protein samples were thoroughly vortex mixed with 10 µL 1% trifluoroacetic acid, and the supernatants were desalted and freeze‐dried after centrifugation for 5 min at 4°C, 8000 g using a C18 SPE column. Subsequently, liquid chromatography‐tandem mass spectrometry (LC‐MS/MS) analysis and data processing were carried out following the previously established procedures.^32^

### 2.13 MitoTracker staining

Mitochondrial distribution of GV oocytes was visualized using MitoTracker Deep Red FM (C1032, Beyotime). Briefly, living oocytes were cultured in an M2 medium containing 100 nM MitoTracker Deep Red FM for 30 min at 37°C, then the Hoechst 33342 was used to counterstain nuclei. After three washes, the oocytes were placed onto cell culture dishes to capture images using an LSM 800 confocal microscope.

### 2.14 Mitochondrial membrane potential assay

Mitochondrial membrane potential (MMP) was measured by the assay kit with JC‐1 (C2003S, Beyotime). Specifically, GV oocytes were incubated in an M2 medium supplemented with JC‐1 for 30 min, followed by three washes. Finally, oocytes were submitted to an LSM 800 confocal microscope to capture images.

### 2.15 Reactive oxygen species and mitochondrial superoxide measurement

Reactive oxygen species (ROS) levels of GV oocytes were detected using a commercial kit (S0033S, Beyotime). Briefly, the GV oocytes were stained in an M2 medium containing 10 µM 2′,7′‐dichlorodihydrofluorescein diacetate (DCFH‐DA) for 30 min at 37°C. Mitochondrial superoxide (MitoSOX) was detected using the Mitochondrial Superoxide indicator (RM02822, Abclonal). Briefly, oocytes were stained using 5 µM MitoSOX indicator for 30 min at 37°C. After staining and washing three times, oocytes were placed onto cell culture dishes to capture images using an LSM 800 confocal microscope.

### 2.16 Quantification of mitochondrial DNA copy number

Mitochondrial DNA (mtDNA) copy numbers were quantified following protocols described in previous studies.^33^, ^34^ In brief, the amplified mtDNA fragments by ND5‐specific primers (Table S4) were cloned into T‐vectors for plasmid purification. Subsequently, a standard curve was generated using purified plasmid which was diluted from 10‐ to 100 000 000‐fold. The amplification efficiency was calculated using the qPCR efficiency calculator from Roche. For DNA extraction, 10 GV oocytes of WT and ZcKO were lysed in 10 µL buffer supplemented with 200 µg/mL proteinase K, 50 mM Tris‐HCl, 0.5% Tween‐20 and 0.1 mM EDTA. Then, 2 µL the sample was used for qRT‐PCR analysis, and the procedure was conducted as previously described.^33^ Finally, the mtDNA copy number of oocytes was determined using the threshold cycle value and standard curve.

### 2.17 ATP content measurement

The total ATP level was quantified using an ATP assay kit (C2003S, Beyotime). In brief, 15 GV oocytes were collected into 20 µL lysis buffer, followed by centrifugation at 12 000 g for 5 min. Subsequently, 10 µL samples and the standard were utilized to measure the optical density value with a microplate reader. Finally, the ATP content in oocytes was determined using a standard curve.

### 2.18 Statistical analysis

All experiments were repeated three times or more, and data were displayed as the mean ± standard deviation (SD). Statistical significance and data visualization were performed using Student's t‐test and GraphPad Prism version 8.3 (GraphPad Software), respectively.

## 3 RESULTS

### 3.1 Oocyte‐specific deletion of *Eif5* causes mouse follicle development defects and infertility

**FIGURE 1:**
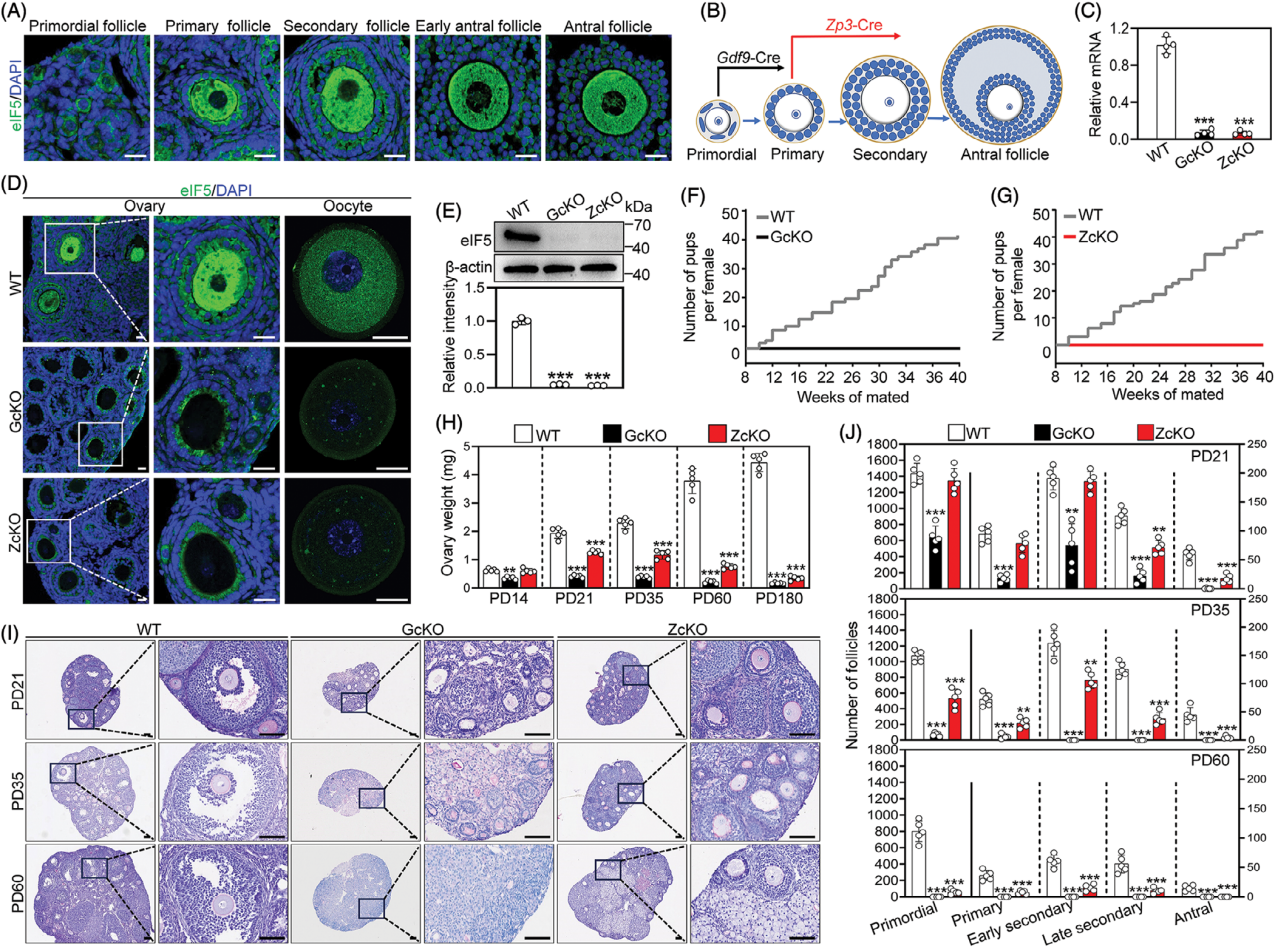
Eukaryotic translation initiation factor 5 (*Eif5*) deletion in oocytes impairs follicle development and female fertility. (A) Representative images of eIF5 staining for follicles at different stages. Cell nuclei were visualized using DAPI. (B) Schematic diagram showing oocyte‐specific deletion of *Eif5* in the primordial follicle by *Gdf9*‐Cre and in the primary follicle by *Zp3*‐Cre. (C) qRT‐PCR results showing *Eif5* mRNA levels in WT, GcKO and ZckO GV oocytes. (D) Immunofluorescence staining of eIF5 in WT, GcKO and ZcKO ovaries (Left) and oocytes (Right) at PD21. (E) Western blot analysis of eIF5 protein levels in WT, GcKO and ZcKO. (F, G) Cumulative numbers of pups per WT (*n* = 6), GcKO (*n* = 5) and ZcKO (*n* = 6) mice during the 40 weeks of a fertility test. (H) Ovarian weights of WT, GcKO and ZcKO female mice, *n* = 5 females each genotype. (I) PAS staining showing ovarian histology of WT, GcKO and ZcKO female mice. (J) Counting of follicle number at different age stages, with *n* = 5 females in each genotype. In each experiment, *n* ≥ 3 biological replicates. Bars indicate the mean  ±  SD. A two‐sided Student's t‐test was used to determine *p*‐values. (**p < 0.01 and ***p < 0.001). Scale bar: 25 µm (A, D) and 100 µm (I).

eIF5 was expressed in both the granulosa cells and oocytes of follicles (Figure 1A). To explore the role of eIF5 in female fertility, we obtained GcKO and ZcKO mice by crossing the *Eif5* ^flox/flox^ female mice with *Gdf9*‐Cre and *Zp3*‐Cre transgenic male mice, respectively (Figure 1B and Figure S1A). qRT‐PCR, IF and Western blot (WB) analysis collectively confirmed that eIF5 was almost completely deleted in all stage oocytes in GcKO ovaries (Figure 1C–E). In ZcKO ovaries, eIF5 was only observed in primordial oocytes (Figure 1D), and only trace amounts of eIF5 were detected in growing oocytes of ZcKO mice (Figure 1C–E). The possible reason is that some residual eIF5 persists in the oocytes and the knockout by *Zp3*‐Cre prevents new eIF5 expression in the oocytes of growing follicles. These results confirm the specificity and effectiveness of *Eif5* deletion in the primordial oocytes by *Gdf9*‐Cre and in the growing oocytes by *Zp3*‐Cre.

Fertility testing confirmed that these two cKO female mice were completely infertile (Figure 1F,G), therefore we first observed the ovarian morphology at different ages. The results showed that the GcKO ovaries were smaller in both size and weight compared to those of WT ovaries at PD14 (Figure 1H,I and Figure S1B,C). Consistently, the results of follicle count exhibited that the number of primary, early secondary (granulosa cells: 2−3 layers) and late secondary follicles (granulosa cells: ≥3 layers) was significantly decreased in GcKO ovaries at PD14, antral follicles were absent in GcKO ovaries at PD21 (Figure 1I and Figure S1D). The ovarian size and weight showed no difference in ZcKO mice at PD14 but became smaller at PD21 (Figure 1H,I and Figure S1B,C). The results of follicle count exhibited that ZcKO ovaries had comparable numbers of primordial, primary and early secondary follicles compared with those of WT ovaries at PD21, but had significantly fewer late secondary and antral follicles (Figure 1J). At PD35, all stages of follicles were significantly decreased in ZcKO mice (Figure 1J). Noteworthy, the number of primordial follicles without *Eif5* deletion was also reduced simultaneously with growing follicle decrease in ZcKO ovaries (Figure 1J). Compared with WT mice, PAS staining showed that the GcKO and ZcKO mice underwent premature ovarian failure as early as PD21 and PD35, respectively, characterized by an absence of mature follicles in the ovaries (Figure 1I). All follicles were completely depleted in both GcKO and ZcKO mouse ovaries at PD180 (Figure S1C,D). In summary, these results indicate that *Eif5* deletion in oocytes impairs follicle development, leading to infertility.

### 3.2 Oocyte‐specific deletion of *Eif5* impairs oocyte developmental competence

**FIGURE 2:**
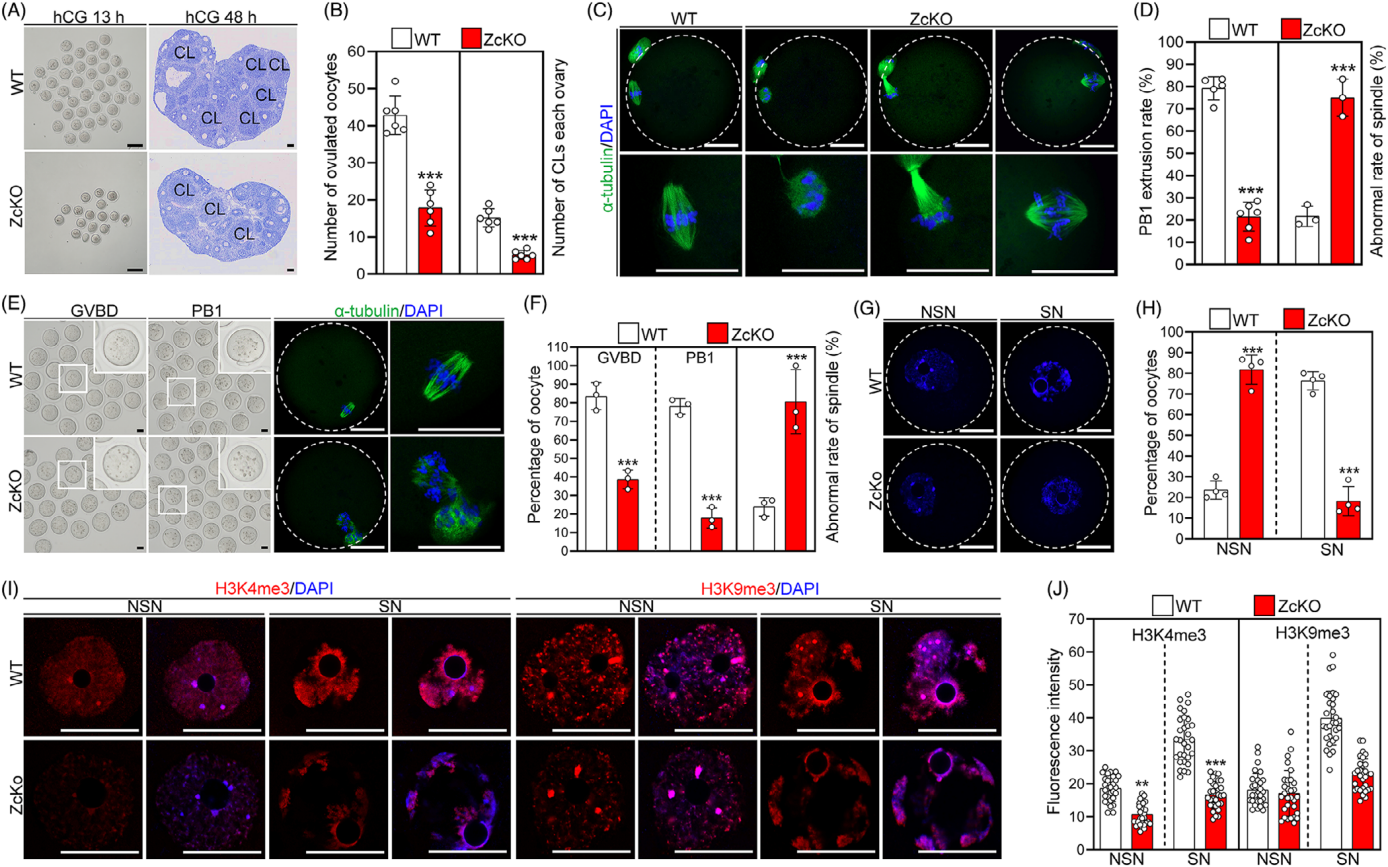
Eukaryotic translation initiation factor 5 (*Eif5*) deletion in oocytes impairs oocyte maturation. (A) Representative images of ovulated oocytes and corpus luteum 13 and 48 h post‐hCG treatment, respectively. (B) Number of ovulated oocytes per female mice and corpora lutea in each ovary, *n* = 6 females for each genotype. (C) α‐tubulin staining showing spindle assembly in superovulated oocytes from WT and ZcKO mice. (D) The percentage of PB1 extrusion (WT: *n* = 206, ZcKO: *n* = 82) and abnormal spindle assembly in superovulated oocytes from WT and ZcKO mice. (E, F) Representative images and the percentage of GVBD (WT: *n* = 92, ZcKO: *n* = 88), PB1 extrusion (WT: *n* = 86, ZcKO: *n* = 67) and abnormal spindle assembly in WT and ZcKO oocytes cultured in vitro. (G) DAPI staining of the oocytes with NSN and SN chromatin configurations in WT and ZcKO mice. (H) The percentage of NSN and SN type oocytes in WT (*n* = 263 oocytes) and ZcKO (*n* = 188 oocytes) mice. (I) Immunofluorescence staining of H3K4me3 and H3K9me3 in WT and ZcKO GV oocytes. (J) Quantification of H3K4me3 (WT: *n* = 30, ZcKO: *n* = 30) and H3K9me3 fluorescence intensity. In each experiment, *n* ≥ 3 biological replicates. Bars indicate the mean  ±  SD. A two‐sided Student's t‐test was used to determine *p*‐values. (**p < 0.01, and ***p < 0.001). Scale bar: 100 µm (A) and 25 µm (C, E, G and I).

The follicle development defects and sterile phenotype in cKO female mice suggest underlying functional deficiencies within the oocytes. ZcKO ovaries contain antral follicles at PD21 but are barely observed at the sexual maturity stage. Therefore, the ZcKO mice at PD21 were used to evaluate oocyte developmental competence. After superovulation treatment, the ZcKO mice ovulated an average of 17 oocytes, while the WT mice ovulated an average of 43 oocytes at PD21 (Figure 2A,B). Consistently, a significant decrease in the number of corpora lutea (CLs) was observed in ZcKO ovaries at 48 h post‐hCG (Figure 2A,B). Only 23.43% of the ZcKO‐ovulated oocytes extruded the first polar body (PB1) (Figure 2D), and these mature oocytes underwent failed fertilization. IF staining of α‐tubulin indicated that 75% of these mature oocytes showed abnormal spindle assembly (Figure 2C,D). Similarly, these functional deficiencies were also reproduced in ZcKO oocytes during in vitro maturation (Figure 2E,F). Furthermore, the fully grown oocytes (FGOs) from the eCG‐primed ZcKO and WT mice at PD21 were also evaluated. DAPI staining revealed that 76.4% of the FGOs exhibited the SN chromatin configuration, while 23.6% of them had NSN chromatin configuration in WT mice (Figure 2G,H). In stark contrast, the percentage of SN versus NSN FGOs was reversed in ZcKO mice (Figure 2G,H). The accumulation of both H3K4 trimethylation (H3K4me3) and H3K9 trimethylation (H3K9me3) is required for chromatin NSN‐SN transition and transcriptional silencing in oocytes.^35^, ^36^ Indeed, IF staining indicated a significant decrease in the intensity of H3K4me3 in both ZcKO NSN and SN oocytes compared to WT oocytes, while the intensity of H3K9me3 significantly decreased only in SN oocytes (Figure 2I,J and Figure S2A). Consistently, the WB analysis also verified significantly lower levels of H3K4me3 and H3K9me3 in ZcKO oocytes relative to WT oocytes (Figure S2B,C). These findings imply that *Eif5* deletion in oocytes impairs nuclear maturation and subsequent meiotic maturation.

### 3.3 Oocyte‐specific deletion of *Eif5* impairs the bidirectional communication between the oocyte and granulosa cells

**FIGURE 3:**
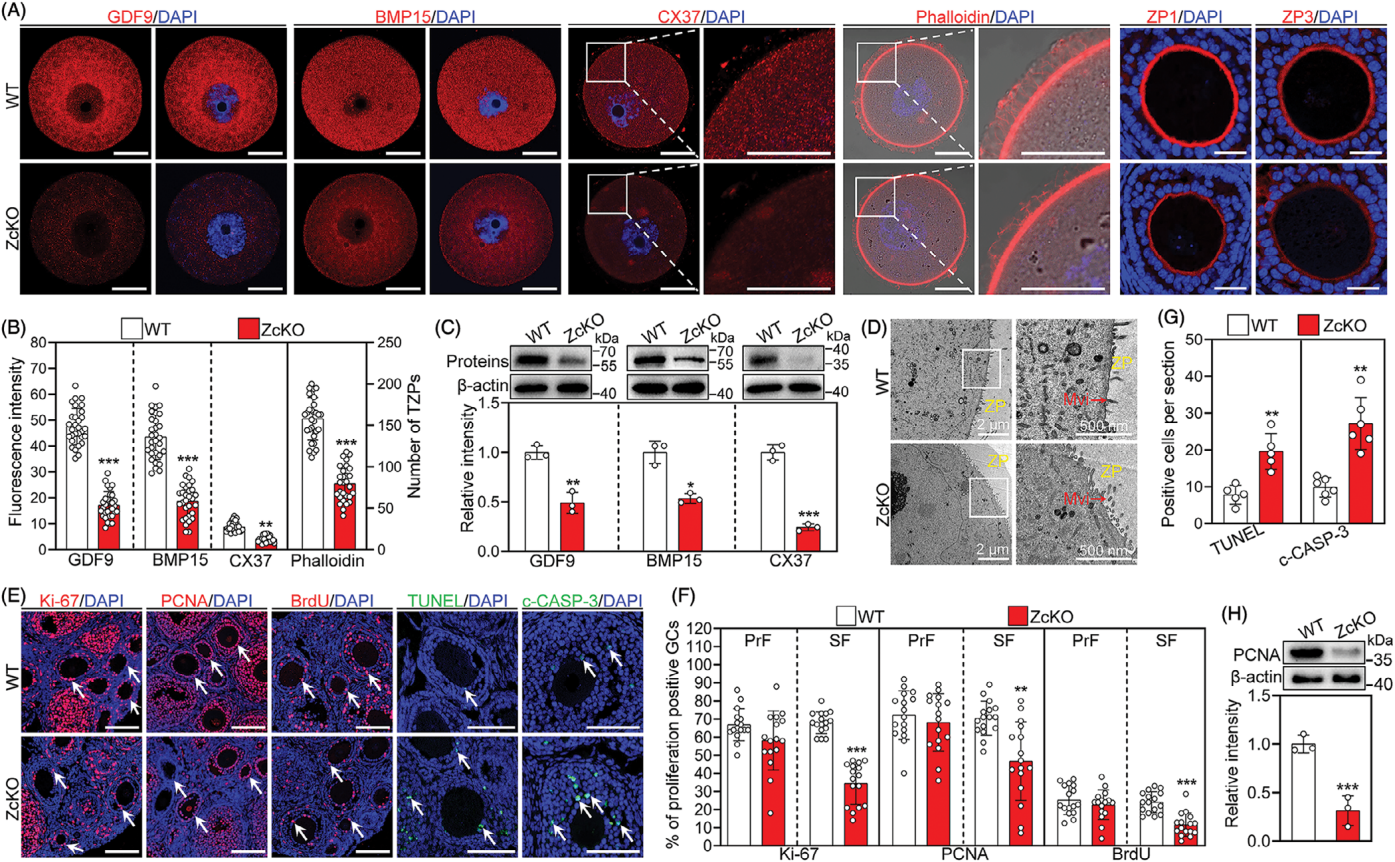
Eukaryotic translation initiation factor 5 (*Eif5*) deletion in oocytes impairs granulosa cell proliferation and induces apoptosis. (A) Immunofluorescence staining of GDF9, BMP15, CX37, Phalloidin, ZP1 and ZP3 in WT and ZckO GV oocytes. (B) Quantification of fluorescence intensity and TZPs number in WT (*n* = 30) and ZckO (*n* = 30) oocytes. (C) Western blot analysis of GDF9, BMP15 and CX37 levels in WT and ZcKO GV oocytes. (D) Transmission electron microscopic images of WT and ZcKO GV oocytes. ZP, zona pellucida. Mvi, microvillus. (E) Immunofluorescence staining of Ki‐67, PCNA, BrdU, TUNEL and c‐CASP‐3 (cleaved Caspase‐3) in WT and ZcKO ovaries at PD21. (F) The percentage of Ki67‐, PCNA‐, and BrdU‐positive granulosa cells in primary follicles (PrF) and secondary (SF) follicles. (G) Quantification of TUNEL‐ and c‐CASP‐3‐positive granulosa cells in WT and ZcKO ovaries at PD21. (H) Western blot analysis of PCNA levels in WT and ZcKO granulosa cells. In each experiment, *n* ≥ 3 biological replicates. Bars indicate the mean  ±  SD. A two‐sided Student's t‐test was used to determine *p*‐values. (*p < 0.05, **p < 0.01 and ***p < 0.001). Scale bar: 25 µm (A) and 100 µm (E).

The bidirectional communication between the oocyte and granulosa cells plays a critical role during oocyte growth and follicle development, which has been confirmed to rely on TZPs, gap junctions and microvilli.^37^ These cell structures facilitate the exchange of nutrients, growth factors and metabolites between granulosa cells and the oocytes.^9^ IF and WB analysis showed significant decreases in the protein levels of growth differentiation factor 9 (GDF9), bone morphogenetic protein 15 (BMP15) and connexin 37 (CX37) in ZcKO GV oocytes relative to WT GV oocytes (Figure 3A–C and Figure S3A). Furthermore, given that the TZPs comprise an actin‐rich central core, TZPs were visualized using phalloidin staining. The results showed that ZcKO GV oocytes displayed significantly decreased F‐actin bundles and disorganized distribution within the ZP area, in stark contrast to the dense and orderly distribution in the WT GV oocytes (Figure 3A,B). The thinner ZPs were observed in ZcKO GV oocytes with IF staining of ZP1 and ZP3 (Figure 3A and Figure S3A). Interestingly, TEM observation showed that oocyte‐derived microvilli were degenerated in ZcKO GV oocytes, while a large number of long and intact microvilli projecting into the perivitelline space in WT GV oocytes (Figure 3D).

Considering the bidirectional communication injury and insufficient expression of oocyte‐secreted factors, the proliferation and apoptosis in granulosa cells were evaluated using IF, WB analysis, qRT‐PCR and TUNEL. The percentages of Ki‐67‐, PCNA‐ and BrdU‐positive granulosa cells were significantly decreased (Figure 3E,F and Figure S3B), and the numbers of TUNEL‐ and c‐CASP‐3‐positive signals were significantly increased in secondary follicles and subsequent stages of ZcKO ovaries relative to those of WT ovaries (Figure 3E,G and Figure S3C). Consistent with these findings, a significant decrease in the mRNA and protein levels of PCNA and/or Ki‐67 in ZcKO granulosa cells was observed (Figure 3H and Figure S3D). Furthermore, the mRNA levels of key genes involved in steroidogenesis, including *Cyp19a1*, *Cyp11a1, Esr2* and *Fshr*, showed a significant decrease in ZcKO granulosa cells (Figure S3D). These findings imply that *Eif5* deletion in oocytes impairs granulosa cell proliferation and induces apoptosis via impairing oocyte‐secreted factors expression and bidirectional communication between the oocyte and granulosa cells.

### 3.4 Oocyte‐specific deletion of *Eif5* alters the oocyte proteome

**FIGURE 4:**
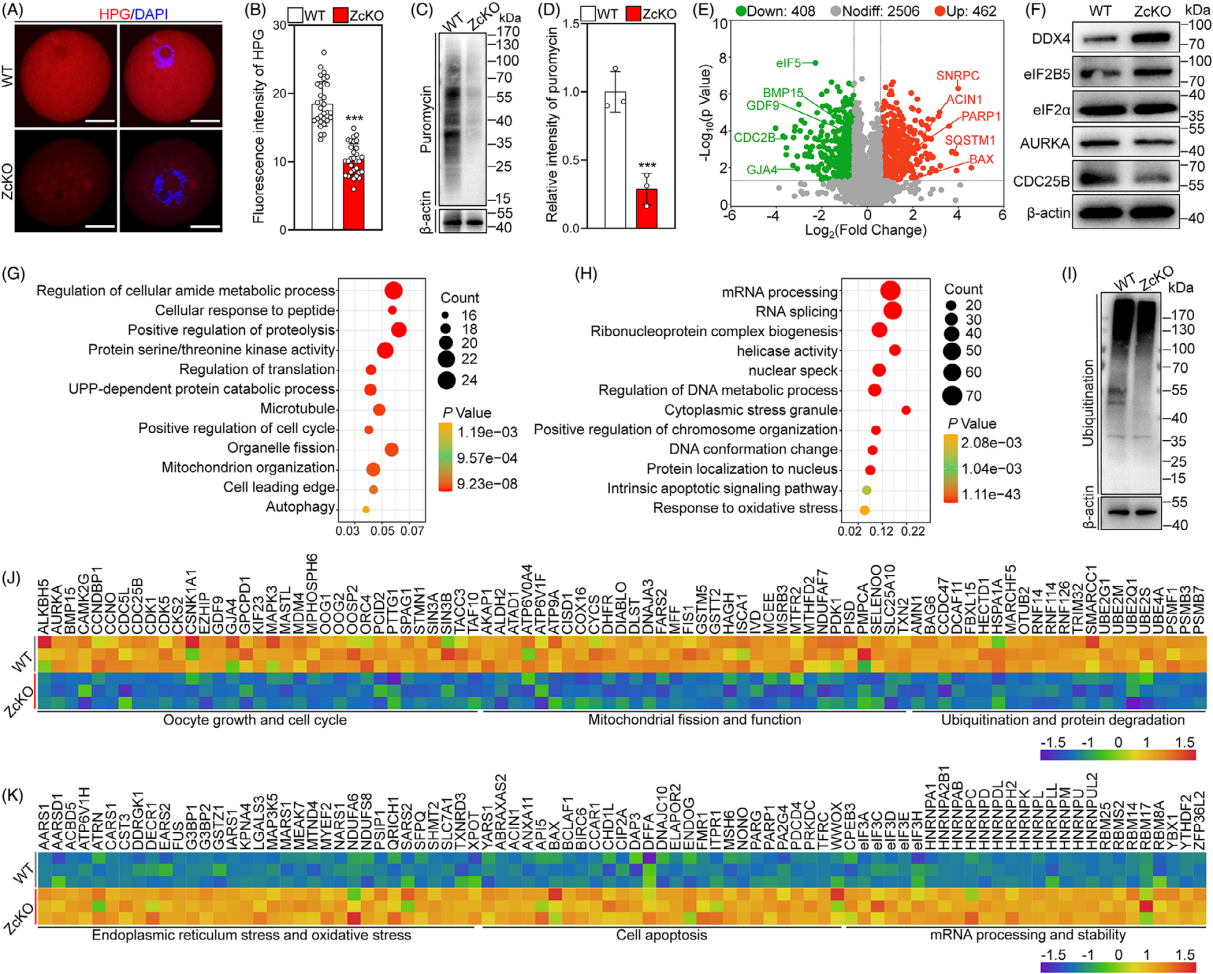
Eukaryotic translation initiation factor 5 (*Eif5*) deletion in oocytes inhibits global protein translation. (A, B) Representative images of HPG fluorescent staining and quantification of fluorescence intensity in WT (*n* = 30) and ZcKO (*n* = 30) GV oocytes. (C, D) Western blot analysis of puromycin incorporation in WT and ZcKO GV oocytes. (E) Volcano diagram illustrating the proteins with differential expression between WT and ZcKO GV oocytes. (F) Western blot analysis of DDX4, eIF2B5, eIF2α, AURKA, CDC25B and β‐actin expression in WT and ZcKO GV oocytes. (G, H) Bubble chart illustrating the GO terms enriched by downregulated and upregulated proteins in ZcKO GV oocytes. (I) Western blot analysis of the total ubiquitination levels in WT and ZcKO GV oocytes. (J, K) Heatmaps illustrating a group of downregulated and upregulated proteins involved in indicated biological processes in WT and ZcKO GV oocytes. In each experiment, *n* ≥ 3 biological replicates. Bars indicate the mean  ±  SD. A two‐sided Student's t‐test was used to determine *p*‐values. (***p < 0.001). Scale bar: 25 µm.

eIF5 plays a crucial role in translation initiation control. Thus, we used two different detection systems to evaluate translational efficiency based on the incorporation of HPG and puromycin, respectively. Compared with WT GV oocytes, the ZcKO GV oocytes exhibited significantly reduced HPG signals and anti‐puromycin intensity, suggesting a decrease in the synthesis of new peptides and proteins (Figure 4A–D). Subsequently, the proteomic landscape of ZcKO GV oocytes was compared with that of WT GV oocytes by LC‐MS. The detected proteins showed a strong correlation between biological replicates (Figure S4A,B). A total of 3376 proteins were identified, and 870 proteins (462 upregulation and 408 downregulation) were differentially expressed by ZcKO GV oocytes (Figure 4E). The changes in protein levels were validated through WB analysis of selected representatives, including DDX4, eIF2B5, eIF2α, AURKA and CDC25B (Figure 4F). Gene Ontology (GO) analysis revealed that the downregulated proteins were involved in the process including oocyte growth and cell cycle, mitochondrial fission and function, as well as ubiquitination and protein degradation (Figure 4G,J). In particular, the downregulated protein levels of GDF9, BMP15 and CX37 could impair the proliferation, glycolysis, tricarboxylic acid (TCA) cycle and cholesterol biosynthesis in granulosa cells (Figure 4J). These proteomic data further confirm the results shown above in Figure 3C. Furthermore, the pronounced downregulation of ubiquitin‐conjugating enzymes (E2), ubiquitin ligases (E3) and proteasome subunits could lead to a reduction in ubiquitination level (Figure 4I,J). Notably, a significant decrease in proteins essential for mitochondrial fission (such as FIS1, MFF, MTFR2) and electron transport chain assembly (such as ATP9A, COX16, CYCS, NDUFAF7) in ZcKO GV oocytes could contribute to mitochondrial dysfunction (Figure 4J). Differing from downregulated proteins, the upregulated proteins mainly participated in the endoplasmic reticulum and oxidative stress, cell apoptosis and mRNA processing and stability (Figure 4H,K). The upregulation of BAX, PDCD4 and SQSTM1 could directly induce oocyte apoptosis (Figure 4K). Collectively, the proteomic analysis not only demonstrated an insufficient synthesis of proteins essential for oocyte growth but also highlighted a significant reduction in mitochondrial fission‐related proteins, suggesting mitochondrial dysfunction in ZcKO GV oocytes.

### 3.5 Oocyte‐specific deletion of *Eif5* causes mitochondrial dysfunction

**FIGURE 5:**
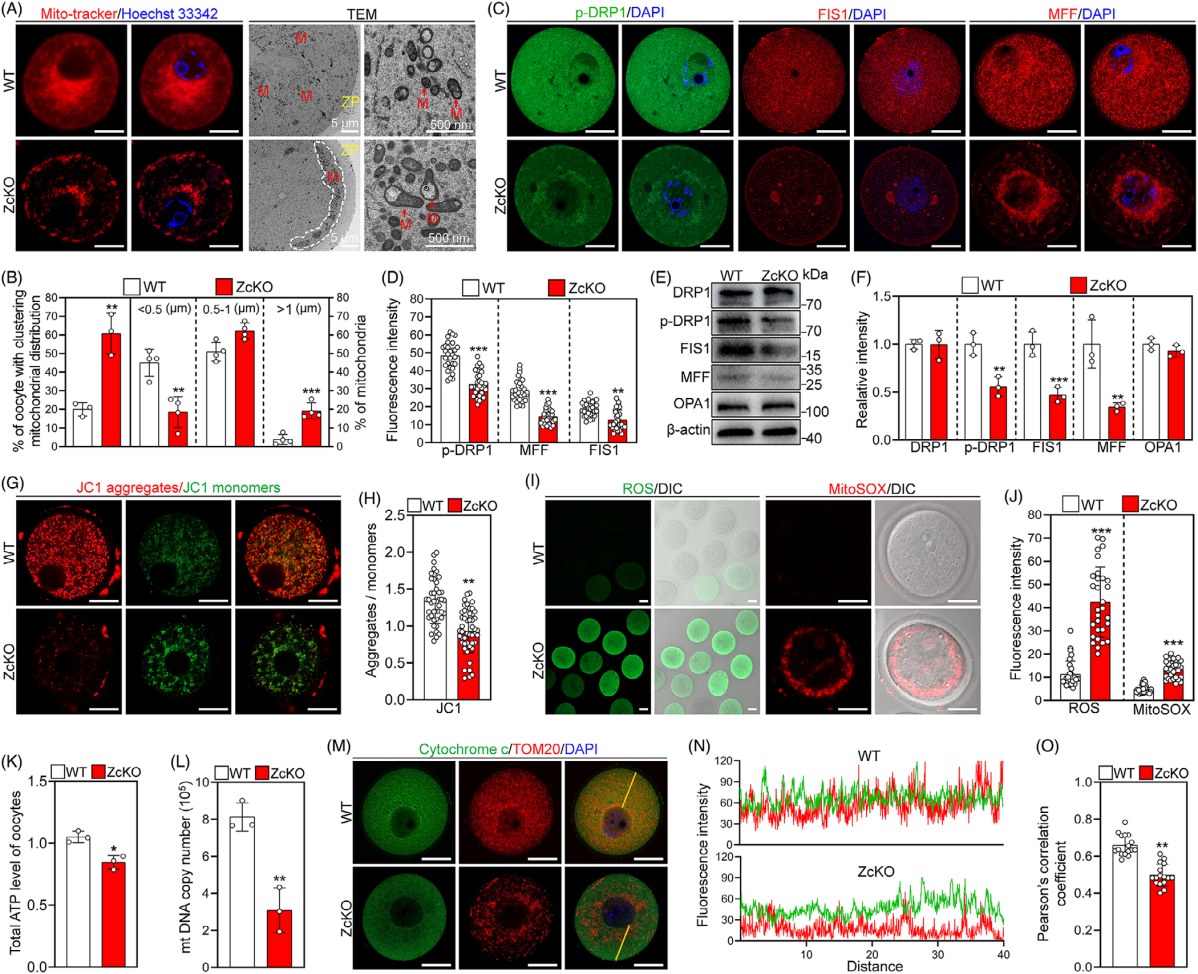
Eukaryotic translation initiation factor 5 (*Eif5*) deletion in oocytes impairs mitochondrial fission and function. (A) Representative images of MitoTracker staining and Transmission electron microscopic (TEM) in WT and ZcKO GV oocytes. ZP, zona pellucida. M, mitochondrion. (B) The percentage of oocytes with abnormal mitochondria distribution (WT: *n* = 30, ZcKO: *n* = 30) and mitochondrial length distribution (WT: *n* = 4, ZcKO: *n* = 4) in WT and ZcKO GV oocytes. (C, D) Immunofluorescence staining of p‐DRP1, FIS1 and MFF, along with quantification of fluorescence intensity in WT and ZckO GV oocytes, *n* = 30 oocytes in each group. (E, F) Western blot analysis of DRP1, p‐DRP1, FIS1, MFF and OPA1 levels in WT and ZcKO GV oocytes. (G) Representative images of JC‐1 staining. (H) Bar chart illustrating the red/green fluorescence ratio of JC‐1 in WT (*n* = 43) and ZcKO (*n* = 48) GV oocytes. (I) Representative images of ROS and MitoSOX detected by DCFH staining and MitoSOX staining in WT and ZcKO GV oocytes. (J) Quantification of ROS and MitoSOX fluorescence intensity in WT (*n* = 30) and ZcKO (*n* = 30) GV oocytes. (K) Measurement of the relative ATP levels in WT and ZcKO GV oocytes (*n* = 15 oocytes in each group). (L) Quantitative analysis of the mtDNA copy number in single WT and ZcKO GV oocytes (*n* = 10 oocytes in each group). (M) Immunofluorescence staining showing co‐localization between Cytochrome c and TOM20 in WT and ZcKO GV oocytes. (N) Fluorescence intensity profiles of Cytochrome c and TOM20 distribution in WT and ZcKO GV oocytes. (O) Quantification of the co‐localization between Cytochrome c and TOM20 using Pearson's correlation coefficient in WT (*n* = 16) and ZcKO (*n* = 16) GV oocytes. The ImageJ software plugin Co‐localization Finder was used to perform the co‐localization analysis. In each experiment, *n* ≥ 3 biological replicates. Bars indicate the mean  ±  SD. A two‐sided Student's t‐test was used to determine *p*‐values. (*p < 0.05, **p < 0.01 and ***p < 0.001). Scale bar: 25 µm.

Mitochondria play a crucial role in oocyte growth and meiotic maturation. Our proteomic results showed a misregulation of proteins associated with mitochondrial fission and function in ZcKO GV oocytes. The mitochondrial distribution and morphology were assessed using MitoTracker staining and TEM analysis. In WT GV oocytes, the mitochondria were discrete and evenly distributed in the ooplasm, while the mitochondria aggregated beneath the cell membrane of ZcKO GV oocytes (Figure 5A). Furthermore, *Eif5* deletion in oocytes led to a significant increase in the percentage of elongated mitochondria and oocytes with clustering mitochondrial distribution compared to WT (Figure 5A,B). Consistent with abnormal mitochondrial morphology, IF staining and WB analysis showed a significant reduction in the levels of mitochondrial fission‐related proteins, including FIS1, MFF and phosphorylated dynamin‐related protein 1 (p‐DRP1) at Ser616 in ZcKO GV oocytes relative to WT GV oocytes (Figure 5C–F). We further assessed the mitochondrial function. JC‐1 staining was utilized to assess MMP. The results showed a significant decrease in fluorescence intensity ratio (aggregates/monomers) in ZcKO GV oocytes compared to WT GV oocytes (Figure 5G,H), representing depolarization of MMP. In addition, the ROS and MitoSOX were significantly increased (Figure 5I,J), and ATP levels and mtDNA copy numbers were significantly decreased in ZcKO GV oocytes (Figure 5K,L). Fluorescence intensity and Pearson's correlation coefficient analysis indicated decreased intensities of Cytochrome c and translocase of outer mitochondrial membrane 20 (TOM20) signals (Figure 5M,N and Figure S5A,B), along with decreased co‐localization between Cytochrome c with TOM20 in ZcKO GV oocytes relative to those in WT GV oocytes (Figure ​5O). These findings suggest that *Eif5* deletion in oocytes leads to mitochondrial dysfunction and oocytes could undergo apoptosis.

### 3.6 Oocyte‐specific deletion of *Eif5* impairs the integrity of transcriptome

**FIGURE 6:**
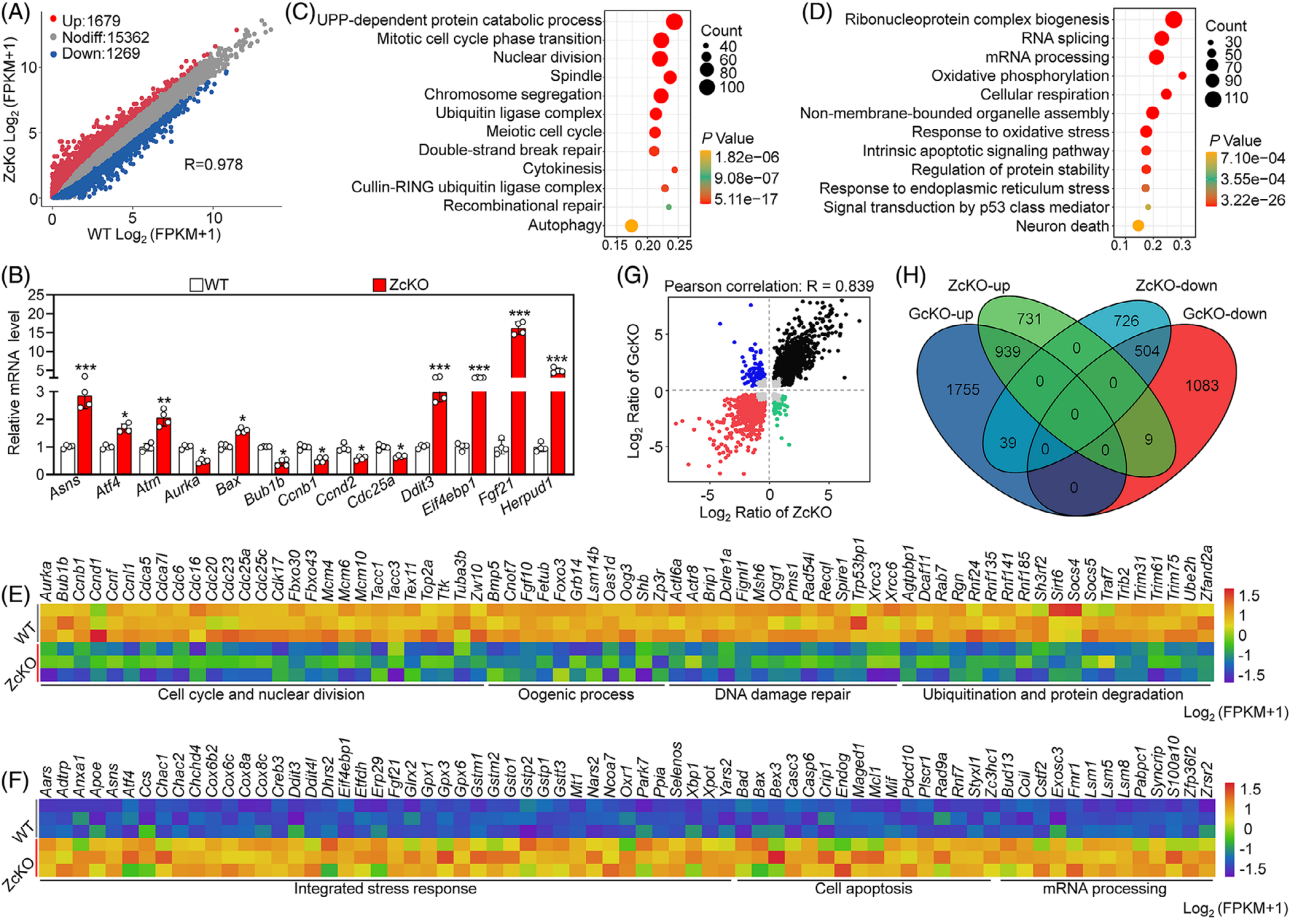
Eukaryotic translation initiation factor 5 (*Eif5*) deletion in oocytes impairs the integrity of the transcriptome. (A) Scatter plot showing the transcripts with differential expression in WT and ZcKO GV oocytes. (B) qRT‐PCR validating the differential transcripts identified by RNA‐seq. (C, D) Bubble chart illustrating the GO terms enriched by downregulated and upregulated transcripts in ZcKO GV oocytes. (E, F) Heatmaps illustrating a group of downregulated and upregulated transcripts involved in indicated biological processes in WT and ZcKO GV oocytes. (G) Scatter plot showing the correlation of differential transcripts between GcKO and ZcKO GV oocytes. (H) Venn diagram showing the relationship of differential transcripts in GcKO and ZcKO GV oocytes. In each experiment, *n* ≥ 3 biological replicates. Bars indicate the mean  ±  SD. A two‐sided Student's t‐test was used to determine *p*‐values. (*p < 0.05, **p < 0.01 and ***p < 0.001).

To assess whether the proteomic alterations caused by *Eif5* deletion were specifically influenced by its involvement in translational control rather than transcriptional modulation, RNA‐seq analysis was performed using oocytes. In ZcKO GV oocytes, 2948 transcripts exhibited differential expression compared with those of WT GV oocytes (1679 upregulation and 1269 downregulation) (Figure 6A). The expressive changes of the representative transcripts were validated using qRT‐PCR (Figure 6B). GO analysis indicated that the downregulated transcripts in ZcKO GV oocytes mainly controlled cell cycle and nuclear division, oogenic process, ubiquitination and protein degradation (Figure 6C,E). These downregulated transcripts in critical processes could impair oocyte growth and follicle development. Particularly, the downregulation of *Foxo3*, *Lsm14b*, *Bub1b* and *Cdc25a* could result in defects in oocyte meiotic maturation and follicle development (Figure 6E). In contrast, the upregulated genes mainly participated in integrated stress response (ISR), cell apoptosis, mRNA processing and oxidative phosphorylation (Figure 6D,F and Figure S6A). In particular, the upregulation of pro‐apoptotic genes (such as *Bax*, *Bad* and *Casp6*) could directly cause the apoptosis of granulosa cells and oocytes (Figure 6F). The upregulation of several mRNA decay‐related genes (such as *Lsm1*, *Zfp36l2* and *Fmr1*) could result in dysregulation of transcript dosage of certain factors key for oocyte growth and maturation (Figure 6F). The changes in the transcriptome of GcKO GV oocytes exhibited a strong positive correlation with those of ZcKO GV oocytes (Figure 6G,H and Figure S6B–G). These results suggest that the reduction in the global translation of oocytes caused by *Eif5* deletion also leads to the downregulation of transcripts related to oocyte growth and ISR activation.

**FIGURE 7:**
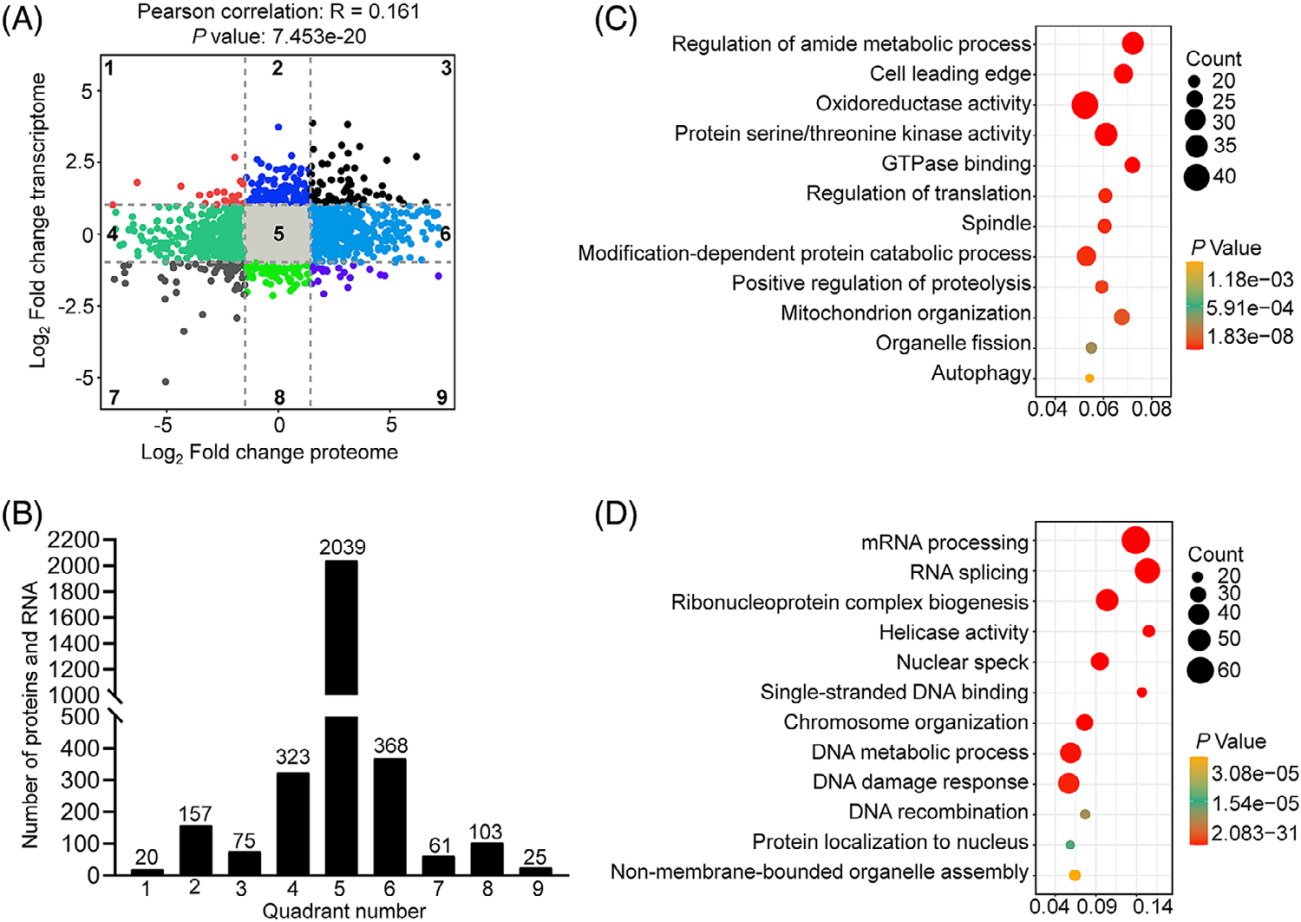
The integration analysis between transcriptome and proteome in ZcKO oocytes. (A) Nine‐quadrant analysis of transcripts and proteins in WT and ZcKO GV oocytes. Dashed lines indicate thresholds for transcripts (FC ≥ 2.0) and proteins (FC ≥ 1.5). (B) Number of transcripts and proteins enriched in nine quadrants. (C, D) Bubble chart showing GO terms enriched by proteins in quadrants 1, 2 and 4 and quadrants 6, 8 and 9.

Subsequently, the integrated analysis of identified the 3171 transcripts and proteins from WT and ZcKO GV oocytes was conducted using a nine‐quadrant diagram (Figure 7A). The number of proteins and transcripts in each quadrant is shown in Figure 7B. Specifically, 2039 proteins in quadrant 5 showed no significant changes at either mRNA or protein levels. 157 proteins in quadrant 2 and 103 proteins in quadrant 8 were differentially expressed at mRNA levels but not at protein levels. The 323 proteins in quadrant 4 and 368 proteins in quadrant 6 were not differentially expressed at mRNA levels but at protein levels. 20 proteins in quadrant 1 and 25 proteins in quadrant 9 displayed a negative correlation between the differentially expressed mRNA and protein levels. In contrast, 75 proteins in quadrant 3 and 61 proteins in quadrant 7 displayed a positive correlation between the differentially expressed mRNA and protein levels, and the enrichment results are shown in Figure S7A,B. Pearson's correlation coefficient analysis indicated that there was no correlation between the differentially expressed proteins and transcripts in *Eif5* deletion oocytes (R = 0.161, Figure 7A), consistent with that of *Eif4e1b* deletion in oocytes.^18^ Proteins in quadrants 1, 2 and 4 were downregulated at the translational level, with enrichment results indicating a term related to oxidoreductase activity, which is crucial for protecting cells from oxidative stress‐induced damage (Figure 7C). Proteins in quadrants 6, 8 and 9 were upregulated at the translational level, with enrichment results highlighting terms related to the DNA damage response and DNA recombination, which is known to be activated in the case of DNA damage (Figure 7D).

### 3.7 Oocyte‐specific deletion of *Eif5* induces DNA damage and apoptosis of oocytes

**FIGURE 8:**
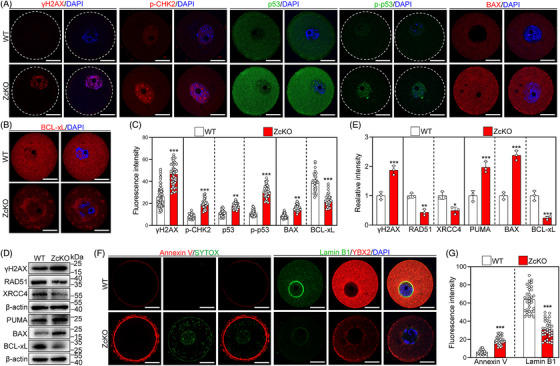
Eukaryotic translation initiation factor 5 (*Eif5*) deletion in oocytes induces DNA damage and oocyte apoptosis. (A, B) Immunofluorescence staining of γH2AX, p‐CHK2, p53, p‐p53, BAX and BCL‐xL in WT and ZckO GV oocytes. (C) Quantification of fluorescence intensity of γH2AX (WT: *n* = 60, ZcKO: *n* = 48), p‐CHK2 (*n* = 30), p53 (*n* = 30), p‐p53 (*n* = 30), BAX (*n* = 30) and BCL‐xL (*n* = 30). (D, E) Western blot analysis of γH2AX, RAD51, XRCC4, PUMA, BAX and BCL‐xL expression in WT and ZcKO GV oocytes. (F) Annexin‐V and Lamin B1 fluorescent staining in WT and ZckO GV oocytes. (G) Quantification of fluorescence intensity of Annexin‐V (WT: *n* = 30, ZcKO: *n* = 30) and Lamin B1 (WT: *n* = 34, ZcKO: *n* = 35). In each experiment, *n* ≥ 3 biological replicates. Bars indicate the mean  ±  SD. A two‐sided Student's t‐test was used to determine *p*‐values. (*p < 0.05, **p < 0.01 and ***p < 0.001). Scale bar: 25 µm.

Excessive ROS and MitoSOX may induce DNA damage and trigger cell apoptosis.^38^ The fluorescence intensity and levels of phosphorylated H2AX (γH2AX) were significantly increased in ZcKO GV oocytes compared to those of WT GV oocytes (Figure 8A,C–E and Figure S8A,C), suggesting DNA damage was increased. In contrast, the levels of DNA repair proteins RAD51 recombinase and X‐ray repair cross‐complementing 4 (XRCC4) were significantly reduced in GV ZcKO oocytes compared to those of WT GV oocytes (Figure 8D,E). Consistent with these, the effectors of the DNA damage response, including phosphorylated CHK2 (p‐CHK2, Thr68), phosphorylated p53 (p‐p53, Ser15) and p53, showed a significant increase in fluorescence intensity in ZcKO GV oocytes compared with those in WT GV oocytes (Figure 8A,C and Figure S8B,C). As expected, the proapoptotic proteins such as PUMA (p53 up‐regulated modulator of apoptosis) and BAX were significantly increased (Figure 8A,C–E), as well as a significant reduction in the levels of anti‐apoptotic protein BCL‐xL (Figure 8B–E). Furthermore, Annexin V‐positive signals were significantly increased, while the fluorescence intensity of Lamin B1 was significantly decreased in ZcKO GV oocytes, suggesting that the oocytes underwent apoptosis (Figure 8F,G). These results indicate that *Eif5* deletion in oocytes induces DNA damage and activates the CHK2‐p53‐PUMA‐BAX apoptotic pathway, resulting in oocyte apoptosis.

## 4 DISCUSSION

**FIGURE 9:**
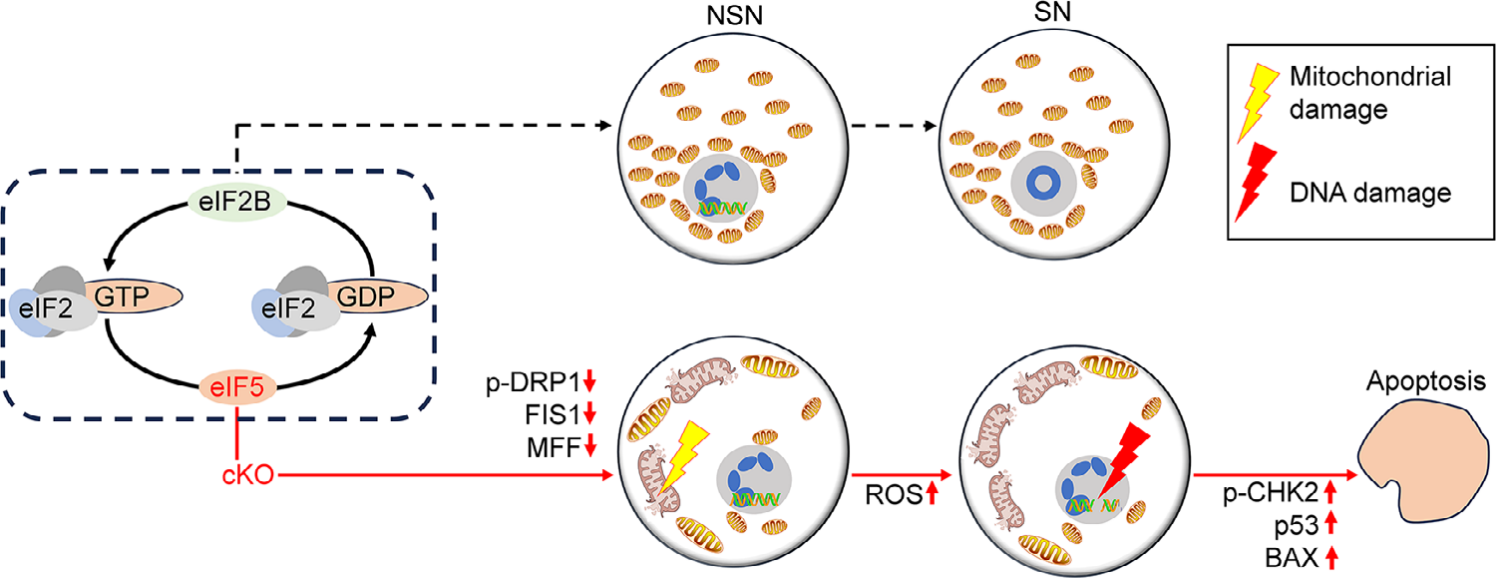
Schematic diagram of eIF5 involved in translation initiation and oocyte growth. Oocyte growth requires massive protein synthesis. eIF5 supports oocyte growth by controlling translation initiation. Generally, eIF5 stimulates hydrolysis of eIF2‐bound GTP to GDP during translation initiation, leading to the release of inactive eIF2·GDP complex. To initiate a new round of translation, the inactive eIF2·GDP complex is recycled to an active eIF2·GTP complex by eIF2B. Depletion of *Eif5* in mouse oocytes impairs the translation of mitochondrial fission‐related proteins (p‐DRP1, FIS1 and MFF), followed by mitochondrial damage. Subsequently, excessive ROS‐induced DNA damage triggers the CHK2‐p53‐BAX pathway, resulting in the apoptosis of oocytes. Illustrations were created with ScienceSlides and BioRender (https://biorender.com/).

The mutations in different translation initiation factors are closely associated with POI by impairing follicle development at different stages. Our research demonstrates that *Eif5* deletion in oocytes impaired the translation of mitochondrial fission‐related proteins. Subsequently, the accumulated excessive ROS could trigger DNA damage and activate the CHK2‐p53‐PUMA‐BAX pathway, resulting in the apoptosis of oocytes within the early‐growing follicles (Figure 9).

In our study, *Eif5* deletion in oocytes caused mitochondrial fission‐related protein translational suppression, mitochondrial dysfunction, ROS accumulation and DNA damage at PD21, followed by oocyte apoptosis of *Eif5* conditional knockout mice at PD35. Mitochondrial fission regulates mitochondrial function, quality and distribution,^39^, ^40^ which is indispensable for oocyte growth and meiotic maturation.^41^, ^42^ Numerous investigations have confirmed that deficiencies in mitochondrial fission‐related proteins lead to mitochondrial dysfunction.^43^, ^44^, ^45^, ^46^ Mitochondrial dysfunction often leads to the excessive accumulation of ROS, followed by DNA damage.^47^, ^48^, ^49^, ^50^ Therefore, the DNA damage caused by *Eif5* deletion in oocytes may be attributed to mitochondrial dysfunction‐induced ROS. DNA damage typically initiates the DNA repair pathways, including HR and non‐homologous end joining (NHEJ).^51^, ^52^ The unrepaired DNA damage will trigger the CHK2‐p53‐dependent apoptotic pathway, ultimately resulting in cell apoptosis.^53^, ^54^ In our study, the key proteins RAD51 in the HR pathway and XRCC4 in the NHEJ pathway were significantly decreased in *Eif5* deletion oocytes, suggesting that *Eif5* deletion‐induced DNA damage can not be repaired and results in oocyte apoptosis. These findings are consistent with prior research indicating that eIF5 deficiencies lead to apoptosis/death in *Drosophila melanogaster* and yeast, characterized by accumulation of DNA damage.^55^, ^56^ Therefore, our study highlights the potential mechanisms of DNA damage and apoptosis caused by eIF5 deficiencies, associated with mitochondrial dysfunction stemming from impaired translation of mitochondrial fission‐related proteins. Enhancing mitochondrial quality and employing antioxidant interventions may be potential therapeutic strategies for POI caused by translation initiation factor mutations.

Oocyte growth requires massive mRNA transcription and protein synthesis.^2^ In our study, the growing follicles displayed development defects and all follicles underwent apoptosis in GcKO mice at PD14 and PD21, respectively. Similarly, the growing follicles displayed development defects and all follicles underwent apoptosis in ZcKO mice at PD21 and PD35, respectively. The continuous development of follicles in the *Eif5* conditional knockout mice could be attributed to the decrease in the ubiquitination levels. The deceleration of eIF5 degradation can continue to maintain protein translation initiation, and the deceleration of the other protein degradation is also conducive to oocyte growth and development in *Eif5* conditional knockout mice. These findings suggest that the residue of eIF5 in the conditional knockout mice is similar to the clinical partial loss of eIF5 function. On the other hand, *Eif5* deletion in oocytes led to the activation of ISR, characterized by the upregulation of activating transcription factor 4 (ATF4)‐target genes and proteins, eIF3 complex and RNA‐binding proteins. The activation of ISR also contributes to the maintenance of oocyte growth and development in *Eif5* conditional knockout mice, as reported in HEK293T cells^57^, ^58^ and neurons.^59^ Interestingly, the primordial follicles without *Eif5* deletion in ZcKO mice also underwent apoptosis simultaneously with the growing follicles apoptosis, consistent with previous reports in *Mfn2*‐ and *Clpp*‐ZcKO mice.^60^, ^61^ This suggests that the presence of growing follicles is beneficial for the survival of primordial follicles. Therefore, follicle development is a continuous and uninterrupted biological process during the female reproductive lifespan. The developmental interruption in growing follicles will result in the premature exhaustion of the primordial follicle.^62^, ^63^, ^64^

The deficiencies of numerous translational regulators can result in disorders in follicle development and oocyte maturation. Translational initiation factor *Eif4e1b* deletion in primary oocytes leads to follicle development arrest at pre‐antral stages but retains oocyte developmental potential.^18^ The translational regulator *Mtor* deletion in primary oocytes impairs the oocyte quality but does not affect follicle development.^22^ In our study, *Eif5* deletion in primary oocytes led to apoptosis of oocytes within the early‐growing follicles. The severe phenotype may be due to the depletion of *Eif5* impairing the global protein translation in oocytes at early developmental stages. Inconsistently, the *Eif4e1b and Mtor* deletion mainly impair the selective translation of specific mRNAs essential for oocyte maturation and the transition from oocyte to embryo.^18^, ^19^, ^22^ These findings suggest that deficiencies in different translation regulators result in different reproductive phenotypes. A recent clinical study reports that patients with Turner syndrome show a lack of a cluster of oogonia with high *EIF5* expression and germ cell apoptosis at 12–13 weeks post‐conception, subsequently developing POI after birth.^65^ Furthermore, the mRNA levels of *EIF5* are decreased in the oocytes of aged women.^66^ These findings suggest that the reduction of eIF5 is closely associated with POI. Our research also supports a previous hypothesis: the POI caused by *EIF2B* gene mutations could be attributed to follicle apoptosis.^67^ Collectively, this evidence offers valuable insights into the pathogenesis and genetic diagnosis of POI. However, these phenotypes and mechanisms still need to be verified using clinical samples in the future.

In conclusion, our study indicates that *Eif5* deletion leads to apoptosis of oocytes within the early‐growing follicles by impaired translation of mitochondrial fission‐related proteins, subsequent excessive ROS accumulation and DNA damage. These findings provide valuable insights into pathogenesis, genetic diagnosis and potential therapeutic targets for POI.

## AUTHOR CONTRIBUTIONS

**Weiyong Wang**: Conceptualization; data curation; formal analysis; investigation; methodology; validation; visualization and writing‐original draft. **Huiyu Liu**: Data curation. **Shuang Liu**: Validation. **Tiantian Hao**: Data curation. **Ying Wei**: Validation. **Hongwei Wei**: Software. **Wenjun Zhou**: Methodology. **Xiaodan Zhang**: Methodology. **Xiaoqiong Hao**: Funding acquisition; project administration and writing‐review & editing. **Meijia Zhang**: Conceptualization; funding acquisition; project administration; resources; visualization; writing‐original draft and writing‐review & editing.

## CONFLICT OF INTEREST STATEMENT

The authors declare no conflict of interest.

## ETHICS STATEMENT

All animal protocols were approved by the Institutional Animal Care and Use Committee of the South China University of Technology (approval number: 2022102).

## Supporting information

Supporting Information

## Data Availability

Raw proteomic data have been deposited in the ProteomeXchange under accession number PXD051435. RNA‐seq data have been deposited in the NCBI Gene Expression Omnibus (GEO) under accession number GSE263785. All data supporting the findings of this study are available within the article and/or the supplementary information. Additional data related to this paper may be requested from the authors.
