# Supplementary material for: Oocyte‐specific deletion of eukaryotic translation initiation factor 5 causes apoptosis of mouse oocytes within the early‐growing follicles by mitochondrial fission defect‐reactive oxygen species‐DNA damage
Source: Clin Transl Med. 2024 Aug 7;14(8):e1791. doi: 10.1002/ctm2.1791 (PMC11306288; doi:10.1002/ctm2.1791)
Supplement: Supplementary file 1 — Supporting Information [file CTM2-14-e1791-s001.docx]

**Supplementary Material**

Oocyte‐specific deletion of eukaryotic translation initiation factor 5 causes apoptosis of mouse oocytes within the early‐growing follicles by mitochondrial fission defect‐reactive oxygen species‐DNA damage

Weiyong Wang^1^, Huiyu Liu^1^, Shuang Liu^1^, Tiantian Hao^1^, Ying Wei^1^, Hongwei Wei^1^, Wenjun Zhou^1^, Xiaodan Zhang^1^, Xiaoqiong Hao^2#^, Meijia Zhang^1#^

1 The Innovation Centre of Ministry of Education for Development and Diseases, the Second Affiliated Hospital, School of Medicine, South China University of Technology, Guangzhou 510006, China

2 Department of Physiology, Baotou Medical College, Baotou 014040, China

Corresponding

Meijia Zhang, The Innovation Centre of Ministry of Education for Development and Diseases, the Second Affiliated Hospital, School of Medicine, South China University of Technology, 382 Outer Ring East Road, Guangzhou Higher Education Mega Center, Guangzhou 510006, Guangdong, China.

E-mail: [zhangmeijia@scut.edu.cn](mailto:zhangmeijia@scut.edu.cn)

Xiaoqiong Hao, Department of Physiology, Baotou Medical College, 31 Jianshe Road, Donghe District, Baotou, 014040, Inner Mongolia, China.

E-mail: haoxiaoqiong@126.com

This file includes:

Supplementary figures and figure legends

Supplementary tables

**
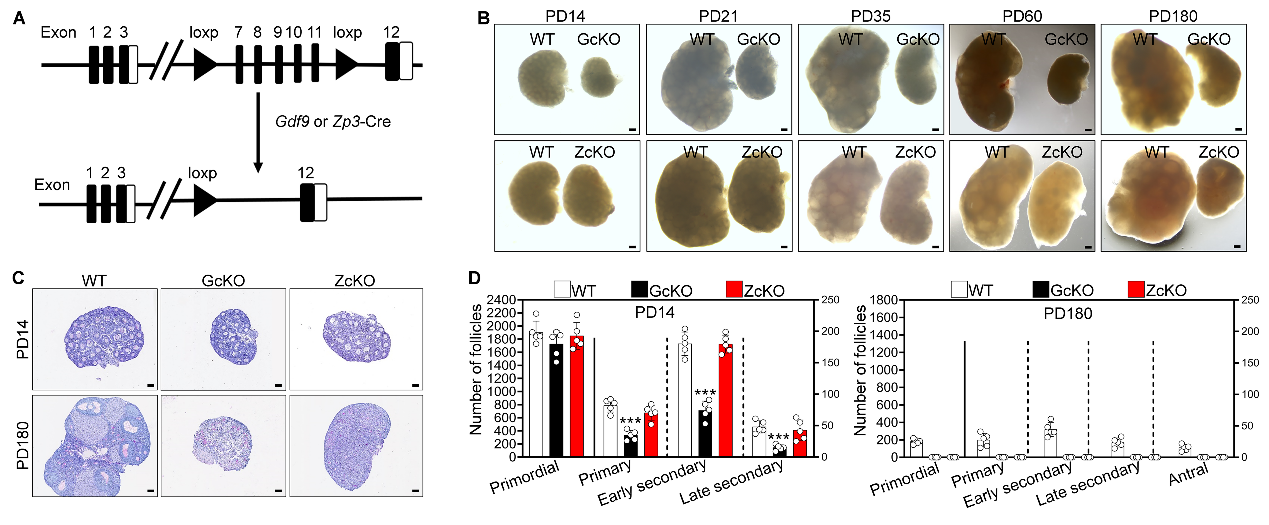
**

**FIGURE S1 Eukaryotic translation initiation factor 5 (*Eif5*) deletion in oocytes impairs follicle development. (A)** Schematic representation of *Eif5* exon 7-11 deletion by *Gdf9*-Cre and *Zp3*-Cre-mediated recombinase in oocytes. **(B)** Representative ovarian images of WT, GcKO and ZcKO female mice at the indicated age. **(C)** PAS staining showing ovarian histology of WT, GcKO and ZcKO mice at the indicated ages. **(D)** Counting of follicle number at different age stages, with *n* = 5 females each genotype. In each experiment, *n* ≥ 3 biological replicates. Bars indicate the mean ± SD. Scale bar: 100 μm.

**
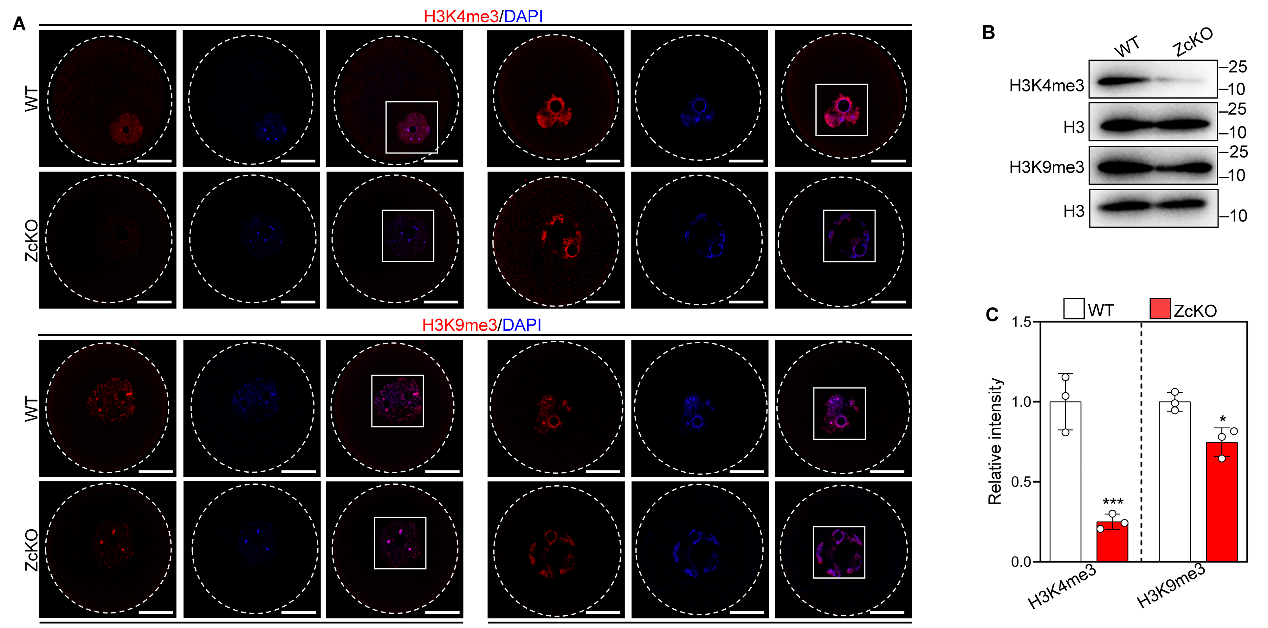
**

**FIGURE S2 Eukaryotic translation initiation factor 5 (*Eif5*) deletion in oocytes impairs histone methylation in oocytes.** **(A)** Immunofluorescence staining of H3K4me3 and H3K9me3 in WT and ZckO GV oocytes. **(B, C)** Western blot analysis of H3K4me3 and H3K9me3 levels in WT and ZcKO oocytes. In each experiment, *n* ≥ 3 biological replicates. Bars indicate the mean ± SD. A two-sided Student’s t-test was used to determine *p*-values. (**p* < 0.05 and ****p* < 0.001). Scale bar: 25 μm.

**
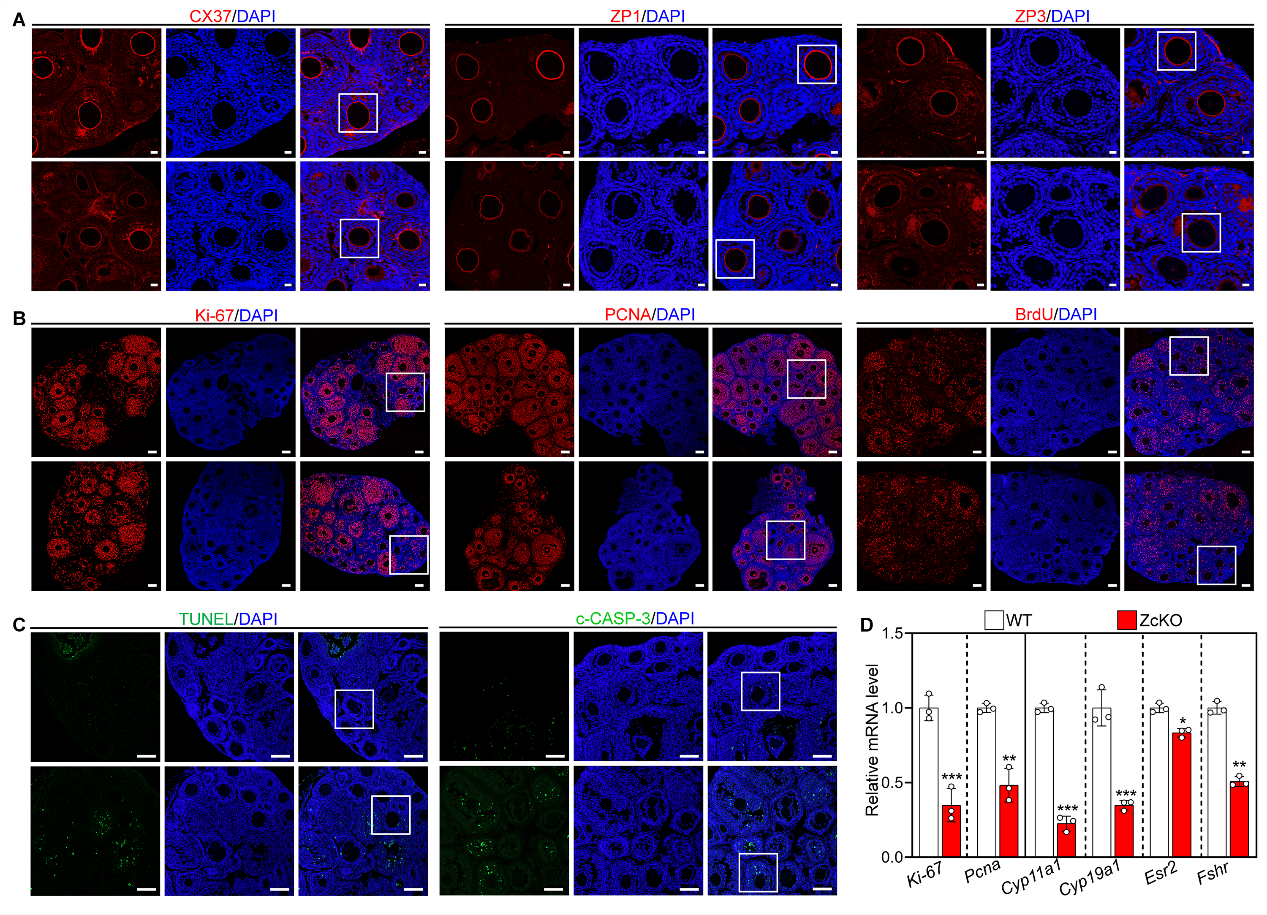
**

**FIGURE S3 Eukaryotic translation initiation factor 5 (*Eif5*) deletion in oocytes impairs the bidirectional communication between the oocyte and granulosa cells. (A)** Immunofluorescence staining of CX37, ZP1 and ZP3 in WT and ZcKO ovaries at PD21. **(B)** Immunofluorescence staining of Ki-67, PCNA and BrdU in WT and ZcKO ovaries at PD21. **(C)** Immunofluorescence staining of TUNEL and c-CASP-3 (cleaved Caspase-3) in WT and ZcKO ovaries at PD21. **(D)** qPCR results showing *Ki-67, Pcna,* *Cyp19a1, Esr2, Fshr* and *Cyp11a1* mRNA levels in WT and ZcKO granulosa cells. In each experiment, *n* ≥ 3 biological replicates. Bars indicate the mean ± SD. A two-sided Student’s t-test was used to determine *p*-values. (**p* < 0.05, ***p* < 0.01, and ****p* < 0.001). Scale bars: 25 μm (A) and 100 μm (B, C).

**
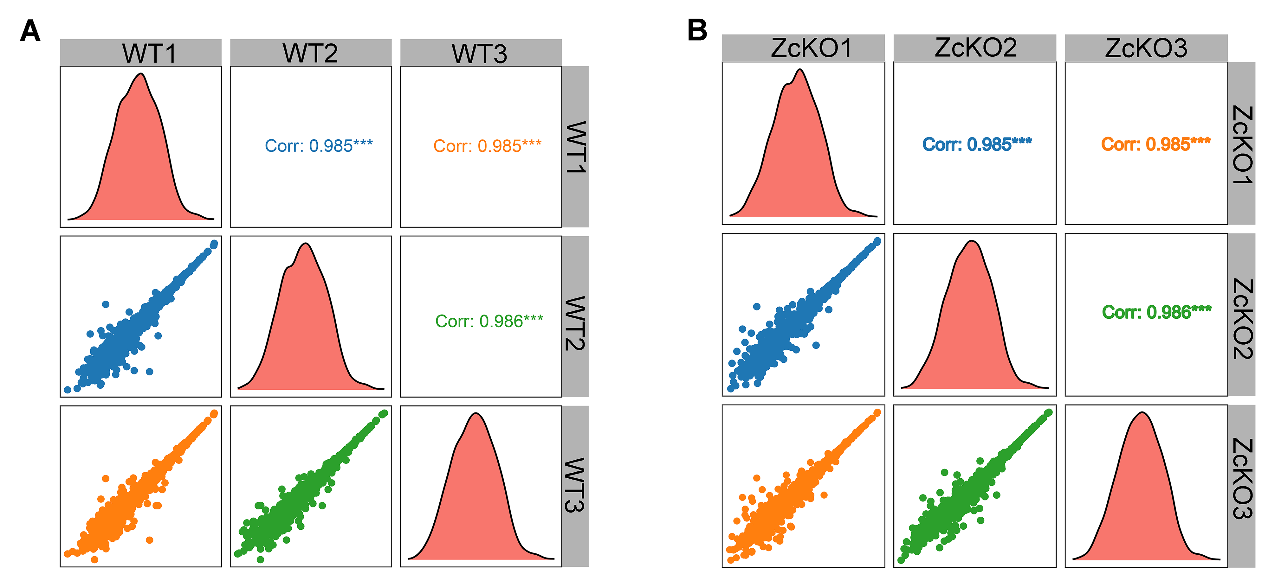
**

**FIGURE S4 The mass spectrometry data were highly correlated in three biological replicates in oocytes.** The collection of three biological replicates in WT **(A)** and ZcKO oocytes **(B)**.

**
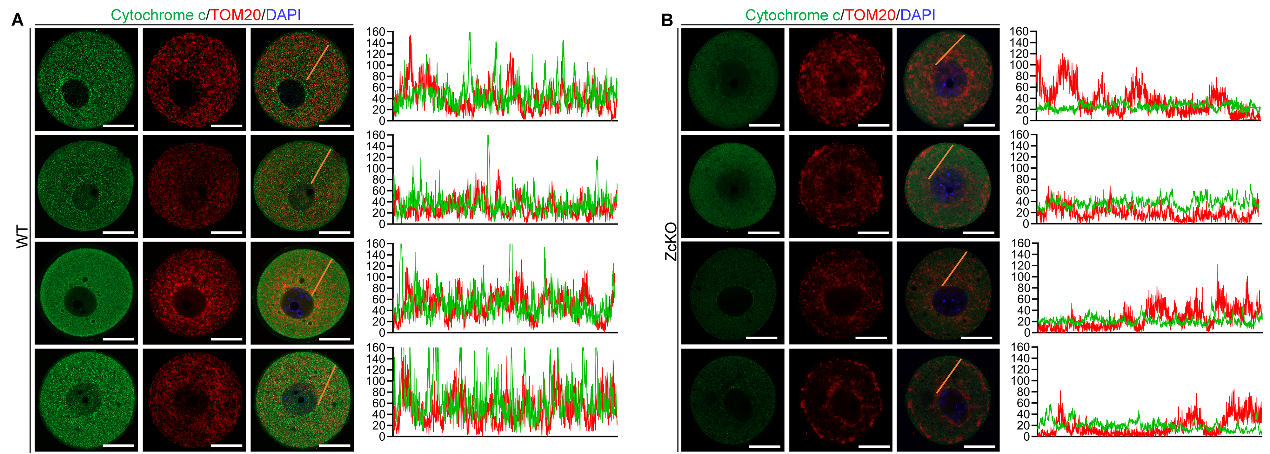
**

**FIGURE S5 The fluorescence profiles of Cytochrome c and TOM20 between WT (A) and ZcKO (B) GV oocytes.**

**
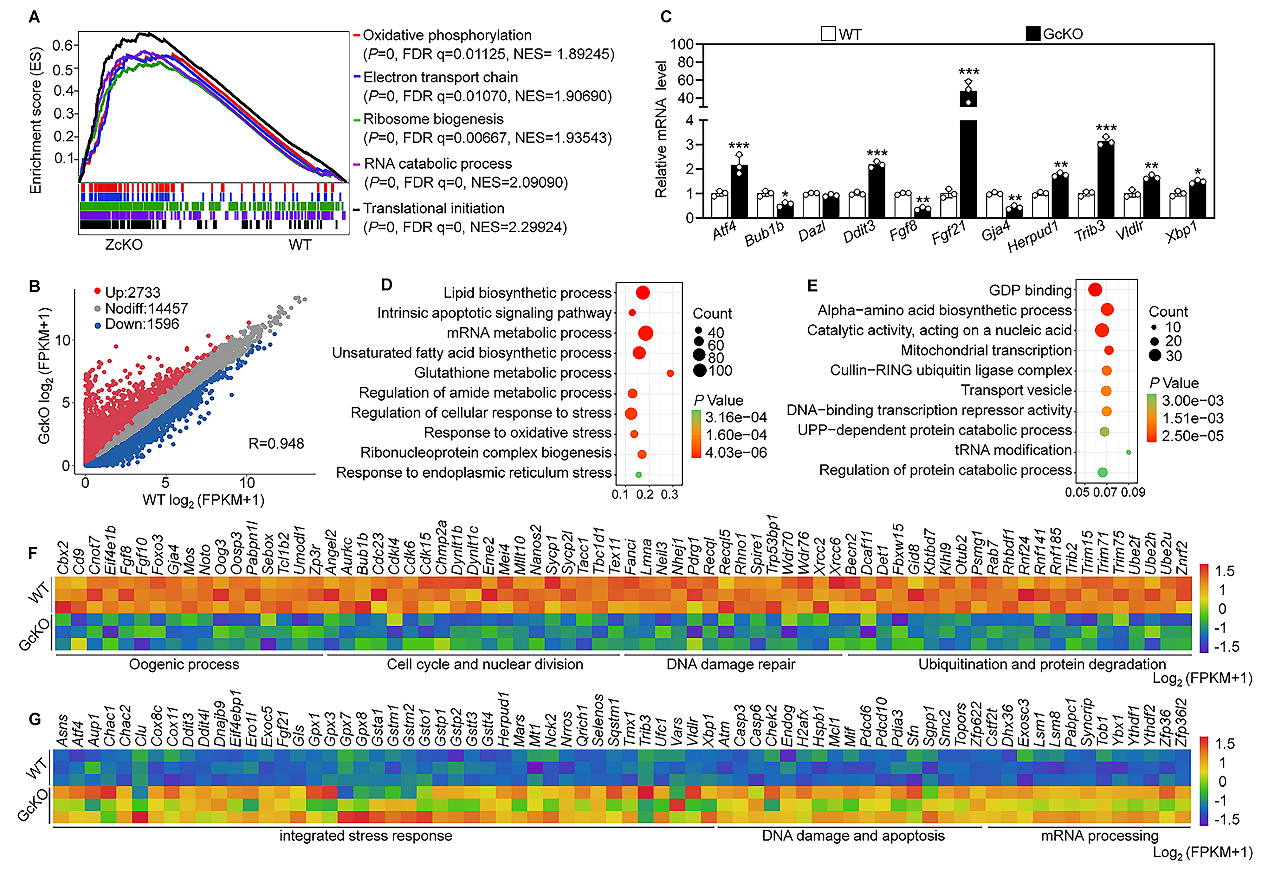
**

**FIGURE S6 Eukaryotic translation initiation factor 5 (*Eif5*) deletion in oocytes impairs the integrity of the transcriptome.** **(A)** GSEA revealing the enrichment of oxidative phosphorylation, electron transport chain, ribosome biogenesis, RNA catabolic process and translational initiation in ZcKO oocytes relative to WT oocytes. **(B)** Scatter plot showing the transcripts with differential expression in WT and ZcKO GV oocytes. **(C)** qRT-PCR validating differential transcripts identified by RNA-seq. **(D, E)** Bubble chart illustrating the GO terms enriched by downregulated and upregulated transcripts in GcKO GV oocytes. **(F, G)** Heatmaps illustrating a group of downregulated and upregulated transcripts involved in indicated biological processes WT and GcKO GV oocytes. In each experiment, *n* ≥ 3 biological replicates. Bars indicate the mean ± SD. A two-sided Student’s t-test was used to determine *p*-values. (**p* < 0.05, ***p* < 0.01, and ****p* < 0.001).

**
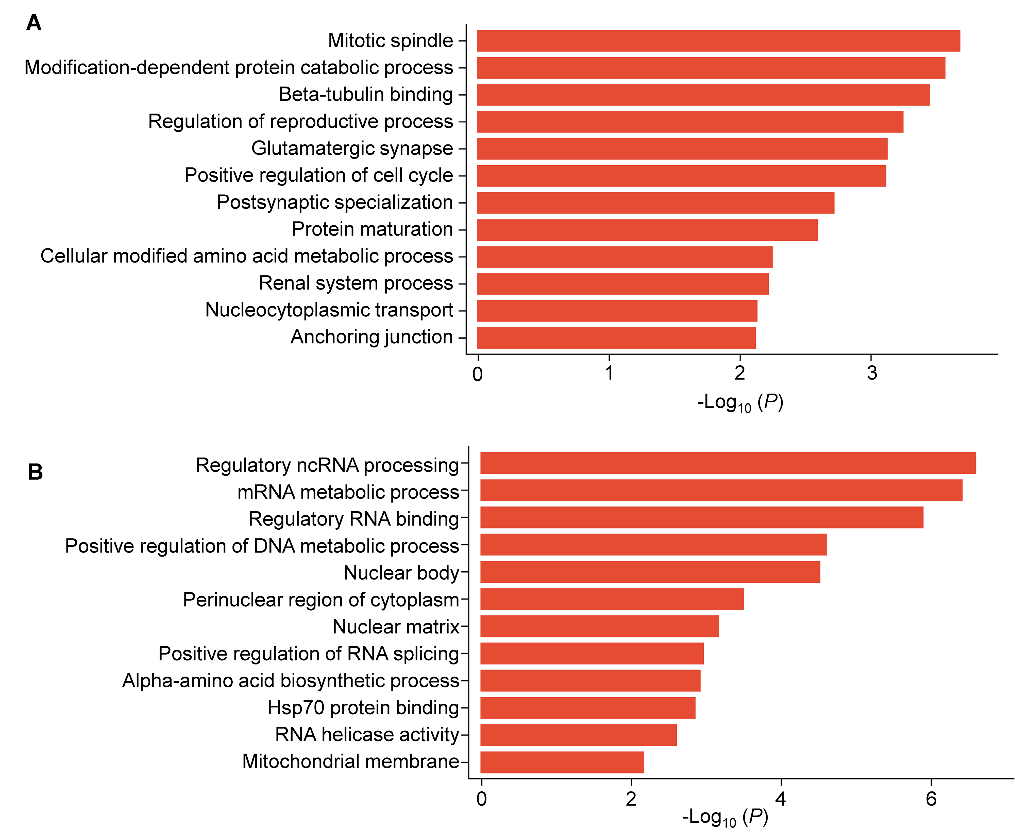
**

**FIGURE S7 The integration analysis between transcriptomic and proteomic in ZcKO oocytes.** Bar chart illustrating the GO terms enriched by proteins in quadrants 3 **(A)** and 7 **(B)**.

**
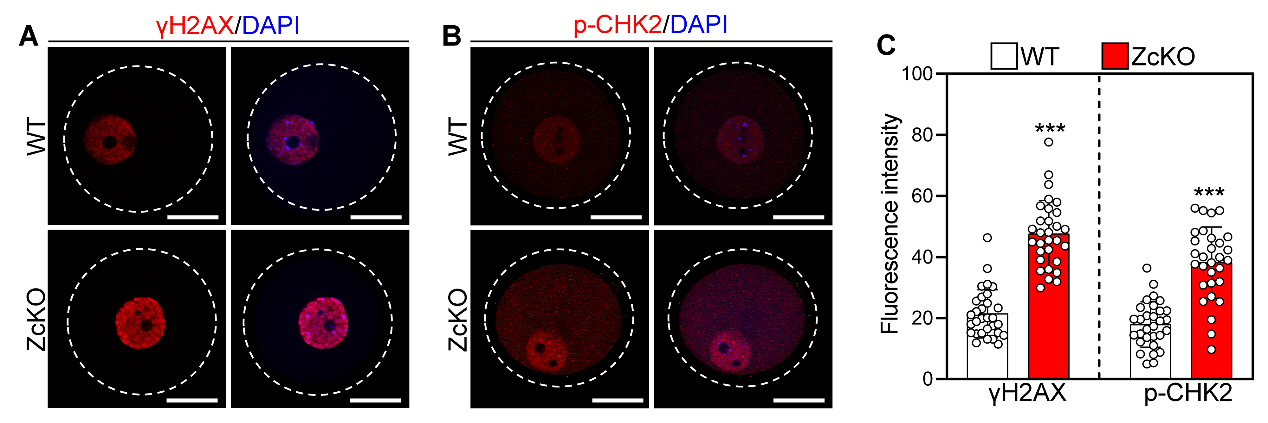
**

**FIGURE S8 Eukaryotic translation initiation factor 5 (*Eif5*) deletion in oocytes activates the DNA damage response pathway. (A, B)** Immunofluorescence staining of γH2AX and p-CHK2 in WT and ZckO oocytes at PD14. **(C)** Quantiﬁcation of fluorescence intensity of γH2AX (WT: *n* = 30, ZcKO: *n* = 30 ) and p-CHK2 (WT: *n* = 30, ZcKO: *n* = 30 ). In each experiment, *n* ≥ 3 biological replicates. Bars indicate the mean ± SD. A two-sided Student’s t-test was used to determine *p*-values. (****p* < 0.001). Scale bar: 25 μm.

**Table S1. Primers for PCR**

| Primer name | Genes targeted | Application | Sequences (5’-3’) |
| --- | --- | --- | --- |
| FRT-F | *Eif5* | Identification of *Eif5* flox | ATGGGGCAAACTACCTGATAC |
| FRT-R |  |  | TGATTAAAACAAAACGACATT |
| Cre-R1 | *Gdf9*-Cre | Identification of *Gdf9*-Cre | AAGAACCTGATGGACATGTTCAG |
| Cre-R2 |  |  | CTGATTCTGGCAATTTCGG |
| Cre-R1 | *Zp3*-Cre | Identification of *Zp3*-Cre | CAGATGAGGTTTGAGGCCACAG |
| Cre-R2 |  |  | TTCTTGCGAACCTCATCACTC |

**Table S2. Primers for qRT-PCR**

| Genes | Forward primers (5’-3’) | Reverse primers (5’-3’) | |
| --- | --- | --- | --- |
| *Eif5* | CGCAGAATGGATGAAATC | GATGAATCAATAATGCCCTC | |
| *Ki-67* | CATTGACCGCTCCTTTAGGT | TCGCCTTGATGGTTCCT | |
| *Pcna* | TTTGAGGCACGCCTGATCC | GGAGACGTGAGACGAGTCCAT | |
| *Cyp19a1* | CATGGTCCCGCAAACTGTGA | GTAGTAGTTGCAGGCACTTC | |
| *Esr2* | TGTGCTATGGCCAACTTCTG | AGTAACAGGGCTGGCACAAC | |
| *Cyp11a1* | AGATCCCTTCCCCTGGCGACAATG | CGCATGAGAAGAGTATCGACGCATC | |
| *Fshr* | TCTCCAACCTACCCAACTTGCA | TATCGGAGACTGGGAAGATTCTG | |
| *Asns* | GCAGTGTCTGAGTGCGATGAA | TCTTATCGGCTGCATTCCAAAC | |
| *Atf4* | CCTATAAAGGCTTGCGGCCA | CACTGCTGCTGGATTTCGTG | |
| *Atm* | GATCTGCTCATTTGCTGCCG | GTGTGGTGGCTGATACATTTGAT | |
| *Aurka* | CTGGATGCTGCAAACGGATAG | CGAAGGGAACAGTGGTCTTAACA | |
| *Bax* | TGAAGACAGGGGCCTTTTTG | AATTCGCCGGAGACACTCG | |
| *Bub1b* | GAGGCGAGTGAAGCCATGT | TCCAGAGTAAAAGCGGATTTCAG | |
| *Ccnb1* | TGCATTCTCTCAGTGCCCTCCACA | AGACAGGAGTGGCGCCTTGGT | |
| *Ccnd2* | CCTTTGACGCAGGCTCCCTTCT | ACCCTGGTGCACGCATGCAAA | |
| *Cdc25a* | ACAGCAGTCTACAGAGAATGGG | GATGAGGTGAAAGGTGTCTTGG | |
| *Ddit3* | CTGGAAGCCTGGTATGAGGAT | CAGGGTCAAGAGTAGTGAAGGT | |
| *Eif4e1b* | GGGGACTACAGCACCACTC | CTCATCGCTGGTAGGGCTA | |
| *Fgf21* | CTGCTGGGGGTCTACCAAG | CTGCGCCTACCACTGTTCC |  |
| *Herpud1* | GCAGTTGGAGTGTGAGTCG | TCTGTGGATTCAGCACCCTTT |  |
| *Dazl* | ATGTCTGCCACAACTTCTGAG | CTGATTTCGGTTTCATCCATCCT |  |
| *Fgf8* | CTTTTGGAAGCAGAGTCCGA | CCATGTACCAGCCCTCGTAC |  |
| *Gja4* | GGGCAAGCAGGCGAGAG | GTCGAGTGTTCCTGGACCTG |  |
| *Trib3* | ACCTTCAGAGCGACTTGTGGG | GCTTGGCCCAAAAAGTCAGG |  |
| *Vldlr* | GGCAGCAGGCAATGCAATG | GGGCTCGTCACTCCAGTCT |  |
| *Xbp1* | CTGAGTCCGCAGCAGGTG | TTCCAGCTTGGCTGATGAGG |  |
| *Rpl19* | CTGAAGGTCAAAGGGAATGTGTTC | TGGTCAGCCAGGAGCTTCTTG |  |

**Table S3. List of primary antibodies used in immune detection**

| Antibody | Catalog Code | | Source | | Host | | | Dilution | | |
| --- | --- | --- | --- | --- | --- | --- | --- | --- | --- | --- |
|  |  |  |  |  |  |  |  | IF | | WB |
| AURKA | | A2121 | | Abclonal | | Rabbit | — | | 1:1000 | |
| BMP15 | | 18982-1-AP | | Proteintech | | Rabbit | 1:100 | | 1:1000 | |
| BAX | | 50599-2-Ig | | Proteintech | | Rabbit | 1:200 | | 1:1000 | |
| BCL-xL | | 2764 | | CST | | Rabbit | 1:200 | | 1:1500 | |
| BrdU | | ab1893 | | Abcam | | Sheep | 1:200 | | — | |
| CDC25B | | sc-56266 | | Santa Cruz | | Mouse | — | | 1:500 | |
| CX37 | | CX37A11-A | | Alpha Diagnostic International | | Rabbit | 1:100 | | 1:1000 | |
| Cytochrome c | | ab65311 | | Abcam | | Mouse | 1:200 | | — | |
| Cleaved Caspase-3 | | 9664 | | CST | | Rabbit | 1:50 | | — | |
| DDX4 | | ab27591 | | Abcam | | Mouse | 1:200 | | 1:1000 | |
| DRP1 | | A2586 | | Abclonal | | Rabbit | — | | 1:1000 | |
| Phospho-DRP1-S616 | | AP1353 | | Abclonal | | Rabbit | 1:200 | | 1:1000 | |
| eIF5 | | A6583 | | Abclonal | | Rabbit | 1:200 | | 1:1000 | |
| eIF2B5 | | A8670 | | Abclonal | | Rabbit | — | | 1:1000 | |
| eIF2α | | 82936-1-RR | | Proteintech | | Rabbit | — | | 1:1000 | |
| FIS1 | | sc-376447 | | Santa Cruz | | Mouse | 1:100 | | 1:1000 | |
| GDF9 | | ab254323 | | Abcam | | Rabbit | 1:200 | | 1:1000 | |
| H3K4me3 | | ab8580 | | Abcam | | Rabbit | 1:200 | | 1:2000 | |
| H3K9me3 | | ab8898 | | Abcam | | Rabbit | 1:200 | | 1:2000 | |
| H3 | | 17168-1-AP | | Proteintech | | Rabbit |  | | 1:2000 | |
| Ki-67 | | 9129s | | CST | | Rabbit | 1:200 | | — | |
| Lamin B1 | | HY-80205 | | MCE | | Rabbit | 1:200 | | — | |
| MFF | | sc-398617 | | Santa Cruz | | Mouse | 1:100 | | 1:500 | |
| OPA1 | | sc-393296 | | Santa Cruz | | Mouse | — | | 1:500 | |
| p53 | | sc-126 | | Santa Cruz | | Rabbit | 1:100 | |  | |
| Phospho-p53-ser15 | | 9284T | | CST | | Rabbit | 1:200 | |  | |
| PCNA | | 2586 | | CST | | Mouse | 1:200 | | 1:1000 | |
| PUMA | | 98672 | | CST | | Rabbit | — | | 1:500 | |
| Puromycin | | A23031 | | Abclonal | | Rabbit | — | | 1:1000 | |
| Phospho-CHK2-T68 | | HY-80799 | | MCE | | Rabbit | 1:200 | | — | |
| RAD51 | | ab133534 | | Abcam | | Rabbit | — | | 1:1000 | |
| TOM20 | | A19403 | | Abclonal | | Rabbit | 1:200 | | — | |
| Ubiquitin | | PTM-5798 | | PTM | | Rabbit | — | | 1:500 | |
| YBX2 | | sc-393840 | | Santa Cruz | | Mouse | 1:100 | | — | |
| XRCC4 | | A1677 | | Abclonal | | Rabbit | — | | 1:1000 | |
| ZP1 | sc-32751 | | | Santa Cruz | | Mouse | 1:100 | | — | |
| ZP3 | sc-398359 | | | Santa Cruz | | Mouse | 1:100 | | — | |
| α-tubulin | ab195887 | | | Abcam | | — | 1:400 | | — | |
| γH2AX | ab206900 | | | Abcam | | Mouse | — | | 1:500 | |
| γH2AX | ab22551 | | | Abcam | | — | 1:300 | | — | |
| β-actin | 4967 | | | CST | | Rabbit | — | | 1:1000 | |

IF: Immunofluorescence; WB: Western blotting

**Table S4. Primers for quantification of mtDNA copy number**

| Genes | Forward primers (5’-3’) | Reverse primers (5’-3’) |
| --- | --- | --- |
| *ND5* | AACCTGGCACTGAGTCACCA | GGGTCTGAGTGTATATATCATGAAGAGAAT |
